# Selection of relevant domains and instruments for the ASAS core set to assess axial spondyloarthritis in clinical practice: a Delphi survey of stakeholders

**DOI:** 10.1016/j.ero.2026.100246

**Published:** 2026-10-07

**Authors:** Marius L. Smits, Victoria Navarro-Compán, Xenofon Baraliakos, Anna Moltó, Elena Nikiphorou, Wilson Bautista-Molano, Philippe Carron, Adrian Ciurea, Jo Davies, Warren Fong, Rodrigo García-Salinas, Laure Gossec, Désirée van der Heijde, David Kiefer, Uta Kiltz, Yu Heng Kwan, Jo Lowe, Marina Magrey, Denis Poddubnyy, Sofia Ramiro, Alexandre Sepriano, Nelly Ziade, Astrid van Tubergen

**Affiliations:** 1 Department of Rheumatology, Maastricht University Medical Centre+, Maastricht, The Netherlands; 2 Care and Public Health Research Institute (CAPHRI), Maastricht University, Maastricht, The Netherlands; 3 Department of Rheumatology, La Paz University Hospital, IdiPAZ, Madrid, Spain; 4 Rheumazentrum Ruhrgebiet, Herne, Germany; 5 Ruhr-Universität Bochum, Bochum, Germany; 6 Université Paris Cité and Université Sorbonne Paris Nord, Centre for Research in Epidemiology and Statistics, INSERM, INRAE, Paris, France; 7 Rheumatology Department, Bichat Hospital, AP-HP. Nord, Paris, France; 8 Centre for Rheumatic Diseases, Faculty of Life Sciences and Medicine, School of Immunology and Microbial Sciences, King’s College London, London, UK; 9 Centre for Education, Faculty of Life Sciences and Medicine, King’s College London, London, UK; 10 Department of Rheumatology, King’s College Hospital NHS Foundation Trust, London, UK; 11 Department of Rheumatology, University Hospital Fundación Santa Fe de Bogotá, Bogotá, Colombia; 12 School of Medicine, Universidad El Bosque, Bogotá, Colombia; 13 Department of Rheumatology, Ghent University Hospital, Ghent, Belgium; 14 Department of Rheumatology and Molecular Immunology and Inflammation Unit, VIB-UGent Centre for Inflammation Research, Ghent University, Ghent, Belgium; 15 Department of Rheumatology, University Hospital Zurich, University of Zurich, Zurich, Switzerland; 16 Axial Spondyloarthritis International Federation (ASIF), London, UK; 17 Department of Rheumatology and Immunology, Singapore General Hospital, Singapore; 18 Office of Education, Duke-NUS Medical School, Singapore; 19 Department of Medicine, Yong Loo Lin School of Medicine, National University of Singapore, Singapore; 20 Hospital Italiano de La Plata, Universidad Nacional de La Plata, La Plata, Argentina; 21 Sorbonne Public Health Institute, INSERM, Sorbonne Université, Paris, France; 22 Rheumatology Department, Pitié-Salpêtrière Hospital, AP-HP, Paris, France; 23 Department of Rheumatology, Leiden University Medical Centre, Leiden, The Netherlands; 24 Programme in Health Services Research and Population Health, Duke-NUS Medical School, Singapore; 25 Centre for Population Health Research and Implementation, SingHealth Regional Health System, Singapore; 26 Case Western Reserve University School of Medicine, University Hospitals, Cleveland, OH, USA; 27 Division of Rheumatology, University of Toronto and Schroeder Arthritis Institute, University Health Network, Toronto, ON, Canada; 28 Department of Rheumatology, Zuyderland Medical Centre, Heerlen, The Netherlands; 29 NOVA Medical School, Universidade NOVA de Lisboa, Lisbon, Portugal; 30 Department of Rheumatology, Hospital de Egas Moniz-ULSLO, Lisbon, Portugal; 31 Faculty of Medicine, Saint Joseph University of Beirut, Beirut, Lebanon; 32 Rheumatology Department, University Medical Centre Hôtel-Dieu de France, Beirut, Lebanon

## Abstract

**Objectives:**

The Assessment of SpondyloArthritis international Society (ASAS) aims to develop a core set for the assessment of axial spondyloarthritis (axSpA) in clinical practice to improve standardisation. The first step is to explore stakeholder preferences regarding candidate domains and instruments used for their assessment.

**Methods:**

A scoping literature review was conducted to inform the development of a Delphi survey comprising 3 planned rounds, with provision for additional rounds. Three stakeholder groups (ASAS members, non-ASAS rheumatologists, and patients with axSpA) participated. The first and second rounds aimed to select domains for the core set, the third round aimed to select instruments, and an additional (fourth) round re-evaluated the inclusion of 1 domain. The selection of domains and instruments considered 2 assessment scenarios: ‘routine’ and ‘extended’ outpatient visits.

**Results:**

The literature review identified heterogeneous candidate domains. Overall, 156 of 201 (78%) ASAS members, 173 non-ASAS rheumatologists, and 167 patients participated in the first Delphi round. Following 4 rounds, 5 domains were selected for routine visits (‘disease activity’, ‘pain’, ‘peripheral manifestations’, ‘extra-musculoskeletal manifestations’, and ‘treatment aspects’) and 4 supplementary domains for extended visits (‘structural damage’, ‘overall functioning and health’, ‘comorbidities’, and ‘mobility’). One recommended instrument was selected for each routine visit domain, alongside several optional instruments. At least 1 recommended or optional instrument was selected for each extended visit domain.

**Conclusions:**

Five domains for routine visits and 4 supplementary domains for extended visits, with corresponding instruments, were selected based on stakeholder preferences to be considered for the ASAS core set to assess axSpA in clinical practice.

**WHAT IS ALREADY KNOWN ON THIS TOPIC**

• Comprehensive disease assessments in axial spondyloarthritis (axSpA) are important to guide treatment choices and optimise patient outcomes in clinical practice.
• Validated guidelines regarding the content of disease assessments in axSpA in clinical settings are limited.

**WHAT THIS STUDY ADDS**

• For ‘routine’ outpatient visits, disease activity, pain, peripheral manifestations, extra-musculoskeletal manifestations, and treatment aspects were selected as relevant domains for assessment in axSpA based on the preferences of worldwide stakeholders.
• For ‘extended’ outpatient visits (eg, annual or periodic review), structural damage, overall functioning and health, comorbidities, and mobility were selected as additional relevant domains.
• Recommended and optional instruments to assess each of these domains were selected.

**HOW THIS STUDY MIGHT AFFECT RESEARCH, PRACTICE OR POLICY**

 

• The selection of relevant domains and instruments represents an important step in the development of the Assessment of SpondyloArthritis international Society core set to guide the assessment of axSpA in clinical practice.
• This core set will contribute to improved standardisation and consistency in the clinical assessment of patients with axSpA, while ensuring the feasibility of this process.

Alt-text: Unlabelled box dummy alt text

## INTRODUCTION

A suitable assessment of axial spondyloarthritis (axSpA) in clinical practice, addressing the current disease status of each patient and its impact, is essential to guide treatment and optimise long-term outcomes [[1], [2], [3]]. Given the heterogeneity of axSpA and the absence of validated guidelines defining a common assessment framework, current follow-up recommendations highlight a broad range of disease-related aspects that may be considered during outpatient visits [[1], [2], [3], [4], [5], [6], [7]]. However, restrictions in time, resources, and the local context of certain healthcare settings may constrain the extent to which comprehensive assessments can be conducted and have contributed to wide variability in how patients are assessed worldwide [[8], [9], [10]]. This underscores the need for standardisation in the follow-up of axSpA. Bringing clarity and consistency to this process would refine workflows for healthcare professionals (HCPs), optimise coherence across clinical practice, provide a more predictable experience for patients, and support effective allocation of time and resources. Furthermore, such improvements could facilitate longitudinal monitoring of health outcomes, enabling smoother process evaluation and ultimately improving quality of care and achieving better treatment outcomes for patients [2,7].

An important tool for standardising the assessment of clinical outcomes is the use of core outcome sets (COSs). COSs are widely used to determine the minimum that should be assessed and reported, comprising domains and corresponding instruments that have been systematically agreed upon by stakeholders in the relevant field [11]. For axSpA research specifically, the Assessment of SpondyloArthritis international Society (ASAS) COS serves as a guide and has strengthened harmonisation of outcome reporting, providing an opportunity to extend similar benefits to clinical practice [[12], [13], [14], [15]]. However, while research core sets are designed to facilitate evidence synthesis and comparison across studies, clinical core sets are intended to support disease monitoring and therapeutic decision-making, necessitating a tailored development approach. Accordingly, a subsequent ASAS initiative aims to establish a dedicated core set for clinical practice.

A core set intended for clinical practice would need to account for several factors. First, the recommended content of assessments needs to holistically reflect the global status of the patient and the impact of axSpA, while aligning with the preferences of all stakeholders. Second, the core set must be feasible to implement in clinical settings, requiring the selective prioritisation of domains and instruments. Finally, the ASAS-European Alliance of Associations for Rheumatology (EULAR) management recommendations and ASAS quality standards propose supplementing routine outpatient visits with a more comprehensive ‘annual review’, which the core set should reflect [1,2].

To inform the development of the core set, the first step of the ASAS-Practice Recommendations for the Assessment of aXIal Spondyloarthritis (ASAS-PRAXIS) project aimed to explore the preferences of stakeholders regarding the relevance of candidate domains for assessment in patients with axSpA in clinical practice and the instruments used for their assessment.

## METHODS

The ASAS-PRAXIS working group was convened in September 2024 to oversee the development of the core set, consisting of 20 rheumatologists and full ASAS members (including 2 representatives from Young-ASAS), 2 patient representatives from the Axial Spondyloarthritis International Federation (ASIF) (JD and JL), and 1 fellow (MLS). Five working group members (VN-C, XB, AM, EN, and AvT) and the fellow formed the steering committee, which led the project. This preparatory study comprised a scoping literature review, followed by a Delphi survey conducted in 3 planned rounds, with provision for additional rounds if indicated.

### Project scope

This study considered assessments in 2 scenarios: ‘routine’ and ‘extended’ outpatient visits. Routine visits were defined as regular, concise consultations in everyday practice at short intervals, and extended visits as more detailed consultations at longer intervals where additional time is available (reflecting annual or periodic reviews, as described in the ASAS quality standards [2]). Extended visits were further conceptualised as an expanded form of routine visits, whereby all domains considered relevant for routine visits would, by definition, also be considered relevant for extended visits. This framework of 2 assessment scenarios is complementary to existing management recommendations and supports the pragmatic assessment of a broad spectrum of disease aspects [1,2].

### Literature review

The scoping literature review was conducted in November 2024 to identify potential domains for assessment in patients with axSpA in clinical practice. This review expanded upon the domains in the ASAS COS for research settings, which formed the foundation of this project [14]. The search strategy presented in Supplementary File S1 was applied in MEDLINE (via PubMed). Considering the limited number of recommendations specifically addressing the clinical assessment of axSpA, the search was broadened to include publications pertaining to any rheumatic and musculoskeletal disease (RMD). Following exclusion of duplicates and publications outside the project scope based on title and abstract screening, the full texts of all remaining publications were reviewed. References of included publications were checked to identify additional relevant publications, after which data were retrieved on the highlighted domains. A list of candidate domains to offer for voting in the Delphi survey was then compiled.

### Participants

Three key stakeholder groups were invited to participate in the Delphi survey: (i) all ASAS members, (ii) non-ASAS member rheumatologists without a specific clinical or research focus on axSpA, and (iii) patients diagnosed with axSpA. This approach incorporated the perspectives of people with lived experiences of axSpA and those involved in their care, improving the applicability of the results across diverse healthcare settings. ASAS members were informed of the forthcoming survey by e-mail and during the ASAS annual meeting in January 2025, and were requested to disseminate the survey to non-ASAS rheumatologists within their centres and surrounding region. Patients were invited through member patient organisations of ASIF, representing various regions worldwide.

Ethical review was commissioned from the Medical Research Ethics Committee of the Academic Hospital Maastricht/Maastricht University in the Netherlands, which determined that the Dutch Medical Research Involving Human Subjects Act was not applicable to this study (METC azM/UM 2024-0421). All participants who were not members of ASAS provided digital informed consent.

### Delphi survey and selection of domains and instruments

The contents of the Delphi rounds were developed and iteratively refined throughout the study by the working group, informed by the candidate domains identified in the literature review. At the beginning of each round, the objectives, corresponding instructions, and any necessary elaboration for that round were communicated to participants. In addition, participants were encouraged to consider the feasibility of assessments when indicating their preferences in the survey, to prevent an overly extensive, and thereby impractical, selection of domains and instruments. The survey was offered to patients in English and Spanish, adapted in plain language with additional explanations where necessary. Development of the patient survey took place in collaboration with patient representatives and native speakers of both languages, who also pilot-tested each round. More details on each of the 3 planned rounds of the Delphi survey, as well as any additional rounds, are presented in Supplementary File S2. Steering committee and working group meetings took place intermittently to evaluate the progress of the rounds and discuss the results, with ensuing actions (eg, determination of the selection criteria for domains and instruments) taken in accordance with existing methodological guidelines for core set development [11,16,17].

The aim of the first planned round was to evaluate the clinical relevance of the candidate domains identified in the literature review, and to collect information from participants on their demographics and healthcare settings. Participants were first asked to indicate whether they considered each of the candidate domains relevant to assess during routine or extended visits, with the option to also suggest new domains. Subsequently, participants ranked up to 5 domains they deemed most relevant during routine visits, and up to an additional 5 domains for extended visits. A weighted scoring system was applied, in which domains were assigned point values according to their rank per participant, with total scores summed across each stakeholder group to generate a prioritised list. The working group intended to consider, for each visit type, domains that were among the 5 highest-ranked by weighted scoring and rated as relevant by ≥70% in all 3 stakeholder groups as (pre)selected. Domains fulfilling these criteria in at least 1 group would be offered for reranking in a second round of voting.

The aim of the second planned round was to further refine and prioritise the selection of candidate domains. Participants were invited to rerank the domains carried forward from the first round. Up to 5 domains could again be ranked for routine visits, and up to an additional 5 for extended visits, with preselected domains from the first round counting towards the total. Importantly, if a domain was available for voting for both routine and extended visits, participants could only include it in their ranking for 1 of the 2 visit types (given that, by default, domains selected for routine visits would be included for extended visits as well). Following this round, the selection of domains by the working group was agreed upon.

The aim of the third planned round was to select instruments to assess the chosen domains. Candidate instruments per domain were compiled by the working group. For domains overlapping with the ASAS COS for research settings, it was agreed to adopt the recommended instruments from the COS [15], with the exception of those considered clinically unfeasible due to the requirement for specific training (ie, Spondyloarthritis Research Consortium of Canada scoring of magnetic resonance imaging of the sacroiliac joints and spine [18], and modified Stoke Ankylosing Spondylitis Spinal Score [19]). Where necessary, instruments from the research COS were adapted to better align with clinical practice. For new domains, the compilation of instruments was informed by expert opinion and relevant literature. Participants were first asked to categorise each candidate instrument as ‘mandatory’, ‘optional’, or ‘not necessary’ for clinical practice. ‘Mandatory’ instruments were considered strongly recommended, and ‘optional’ instruments were regarded as supplementary and to be applied at the discretion of the rheumatologist based on the clinical context. Next, for each instrument rated as ‘mandatory’ or ‘optional’, participants indicated their likelihood of implementing it in clinical practice on a 0 to 10 numerical rating scale. Alternative instruments could also be suggested. The working group intended to recommend an instrument if ≥70% of participants rated it as ‘mandatory’ and the mean implementation score was ≥7. An instrument would be classified as optional if ≥70% rated it as ‘mandatory’ or ‘optional’ and the mean implementation score was ≥7.

All rounds allowed a minimum response period of 2 weeks, with at least 1 reminder. Nonparticipation in earlier rounds did not preclude being invited for subsequent rounds [20]. When completing subsequent rounds, participants were provided with a summary of the aggregated results from all stakeholder groups, along with their own previous responses (if available). Surveys were developed using Qualtrics (Provo, UT), and data were analysed descriptively. All survey responses were processed anonymously, and results were reported in consideration of the Delphi studies in social and health sciences - recommendations for an interdisciplinary standardized reporting (DELPHISTAR) guidelines [21].

## RESULTS

### Literature review

The search strategy identified 1312 publications (Supplementary Fig S1). Following deduplication and screening of all titles and abstracts, 1237 publications were excluded. A further 30 publications were excluded after full-text screening due to irrelevance to this project. Consequently, 45 articles from the search were included alongside 6 from cross-reference checking for additional articles with specific relevance to axSpA, whereby a total of 51 publications fulfilled the eligibility criteria. The data extracted from the included publications, as summarised in Supplementary File S3, represented a heterogeneous range of aspects that may be assessed in RMDs based on the perspectives of various stakeholders. Twenty candidate domains were derived from these data, which are presented along with their definitions in Supplementary File S4. The candidate domains were discussed during the ASAS annual meeting in January 2025, which gave input for the first Delphi round.

### Participants

The first Delphi round was completed by 156 of 201 (78%) ASAS members, 173 non-ASAS rheumatologists, and 167 patients, whose characteristics are presented in Supplementary File S5. The second and third rounds were completed by 154 of 201 (77%) and 142 of 201 (71%) ASAS members, and by 99 of 173 (57%) and 82 of 173 (47%) non-ASAS rheumatologists who participated in the first round, respectively. The second round was completed by 92 of 167 (55%) patients who participated in the first round; patients did not participate in the third round due to its technical nature. ASAS members and non-ASAS rheumatologists originated from 46 countries, and patients from 17 countries, with global representation spanning all continents (Supplementary Fig S2). In addition, a broad range of healthcare settings were represented, including university hospitals, general hospitals, and private practices. The characteristics of responders and nonresponders to subsequent rounds were largely comparable, although slightly greater attrition was observed among non-ASAS rheumatologists and patients from South America and non-ASAS rheumatologists working in private practice (Supplementary Files S6-S8).

### Selection of domains

Two versions of the first Delphi round were distributed. The original version with 20 candidate domains was completed by ASAS members and a first group of non-ASAS rheumatologists (n = 116/173). The steering committee intermittently reviewed all participant suggestions for new candidate domains and concluded that 2 additional domains should be considered (Supplementary File S4). A revised version of the survey, featuring the expanded list of 22 candidate domains, was therefore constructed and circulated to patients and a second group of non-ASAS rheumatologists (n = 57/173).

Altogether, 2 domains fulfilled the prespecified selection criteria across all 3 stakeholder groups in the first round and were preselected for routine visits: ‘disease activity’ and ‘pain’ (Fig 1; Supplementary File S9). One domain was preselected for extended visits: ‘structural damage’. Domains fulfilling the selection criteria in at least 1 group were offered for reranking in the second round, comprising 7 domains for routine visits, and 8 for extended visits. Of note, the domains fulfilling the selection criteria were identical between non-ASAS rheumatologists who completed the original and revised versions of the survey, except that, for extended visits, ‘self-management’ replaced ‘physical functioning’ among the 5 highest-ranked domains in the latter group (Supplementary File S9). Both domains were therefore carried forward to the second round. Furthermore, the ‘patient-specific factors’ domain, despite being highly ranked by patients, was excluded at this stage following discussions, as it was deemed more appropriate as an overarching principle to be accounted for during all consultations rather than a standalone domain to be assessed. The results of the first round were presented and discussed at the ASAS mid-year meeting in June 2025.

**Figure 1:**
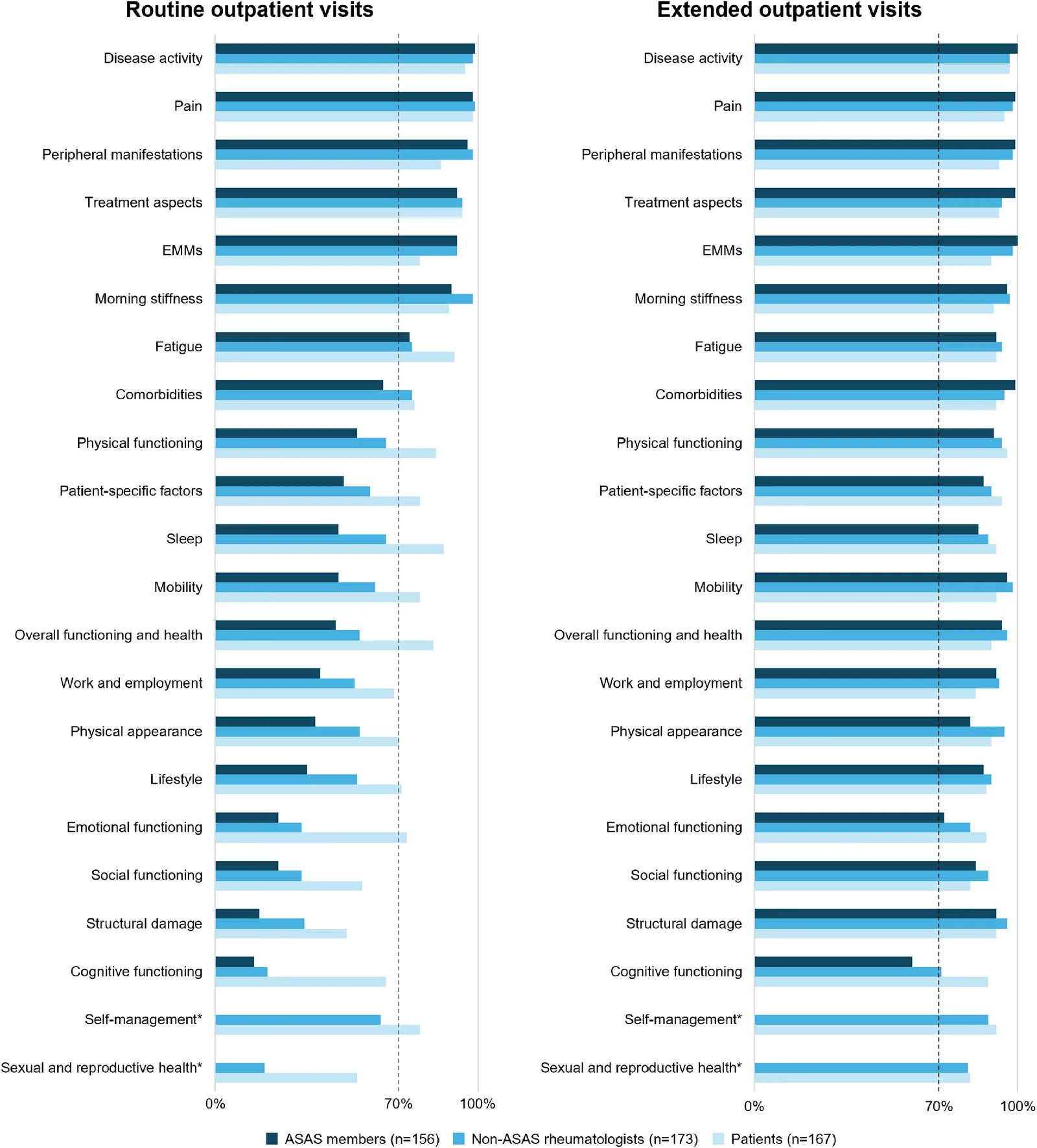
Percentage of participants per stakeholder group who considered each candidate domain relevant for assessment in clinical practice in the first Delphi round. The bars indicate the percentage of participants who rated each domain as relevant, with the 70% cut-off denoted by the dashed line. *Domain was added later to a revised version of the first Delphi round based on participant suggestions. Results presented are therefore only based on participants who completed the revised version (57/173 participating non-ASAS rheumatologists and all 167 patients). ASAS, Assessment of SpondyloArthritis international Society; EMM, extra-musculoskeletal manifestation.

After the second Delphi round, higher concordance in the domains prioritised by each stakeholder group was observed (Fig 2A,B). As such, the results for ASAS members and non-ASAS rheumatologists were henceforth aggregated. Following discussions on the results of the second round, the working group adapted the selection criteria to ensure a comprehensive set of included domains. The adjusted criteria required that a domain was among the 5 highest-ranked by weighted scoring and included in the rankings of >50% of participants in at least 1 stakeholder group, based on the combined results for both visit types (cumulative results are presented in Fig 2C and Supplementary File S10). Combined results were used to prevent underestimating the priority of domains that were available for voting for both routine and extended visits, as each participant could select these domains for only 1 visit type. Accordingly, 3 additional domains were selected for routine visits: ‘peripheral manifestations’, ‘extra-musculoskeletal manifestations’ (EMMs), and ‘treatment aspects’, and 2 for extended visits: ‘overall functioning and health’ and ‘comorbidities’ (Fig 3).

**Figure 2:**
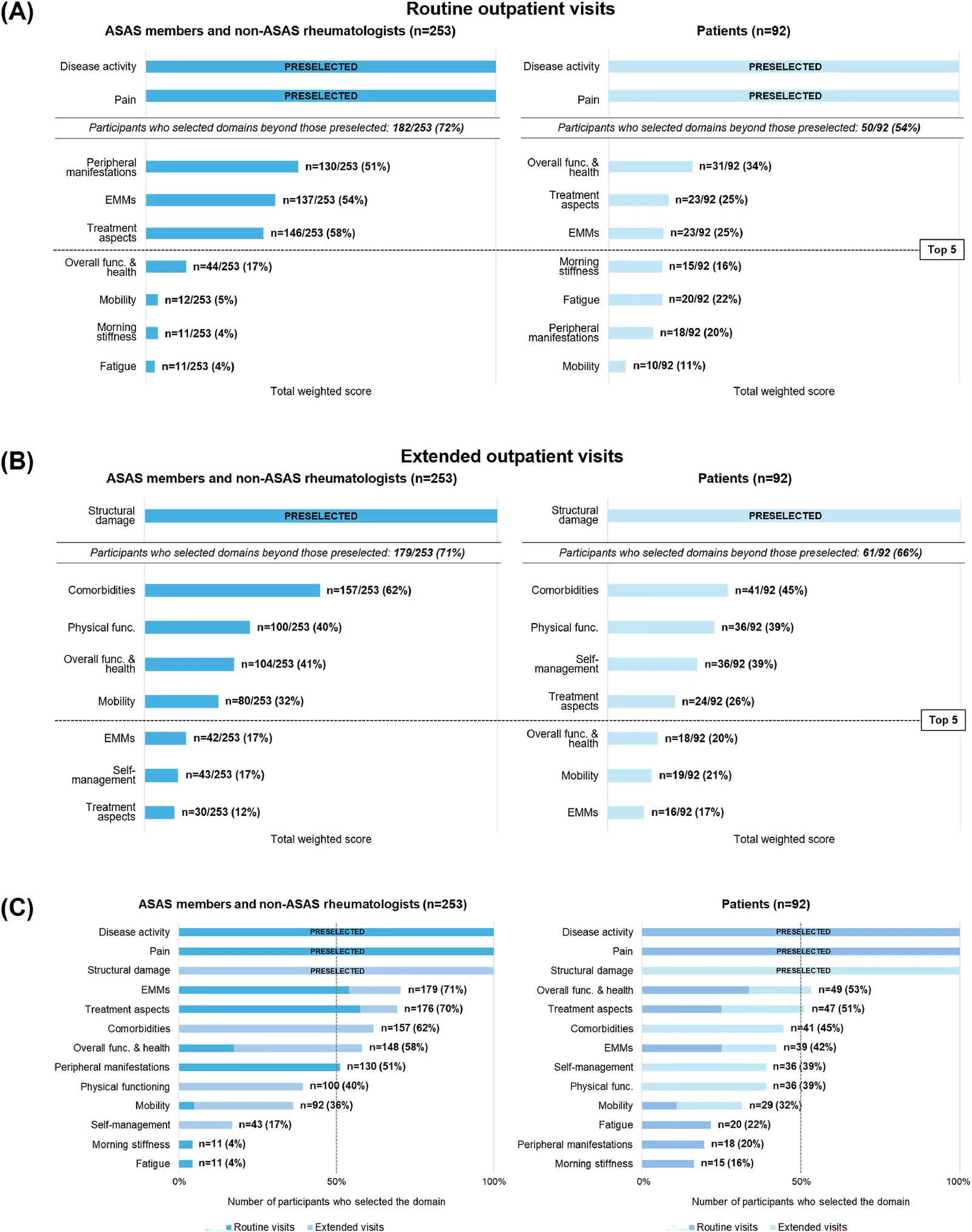
Ranking of domains per stakeholder group in the second Delphi round based on relevance for assessment in clinical practice during (A) routine outpatient visits and (B) extended outpatient visits, and (C) the cumulative number (percentage) of participants who included each domain in their rankings for either visit type. (A and B) The weighted score assigned to each domain is presented relative to the maximum possible score, and the 5 highest-ranked domains are denoted by the dashed line. The proportion of participants who included each domain in their ranking is indicated next to the bars. (C) The cumulative proportion of participants who included each domain in their ranking, irrespective of which visit type the domain was ranked for, is presented, with the 50% cut-off denoted by the dashed line. ASAS, Assessment of SpondyloArthritis international Society; EMM, extra-musculoskeletal manifestation; Func, functioning.

**Figure 3:**
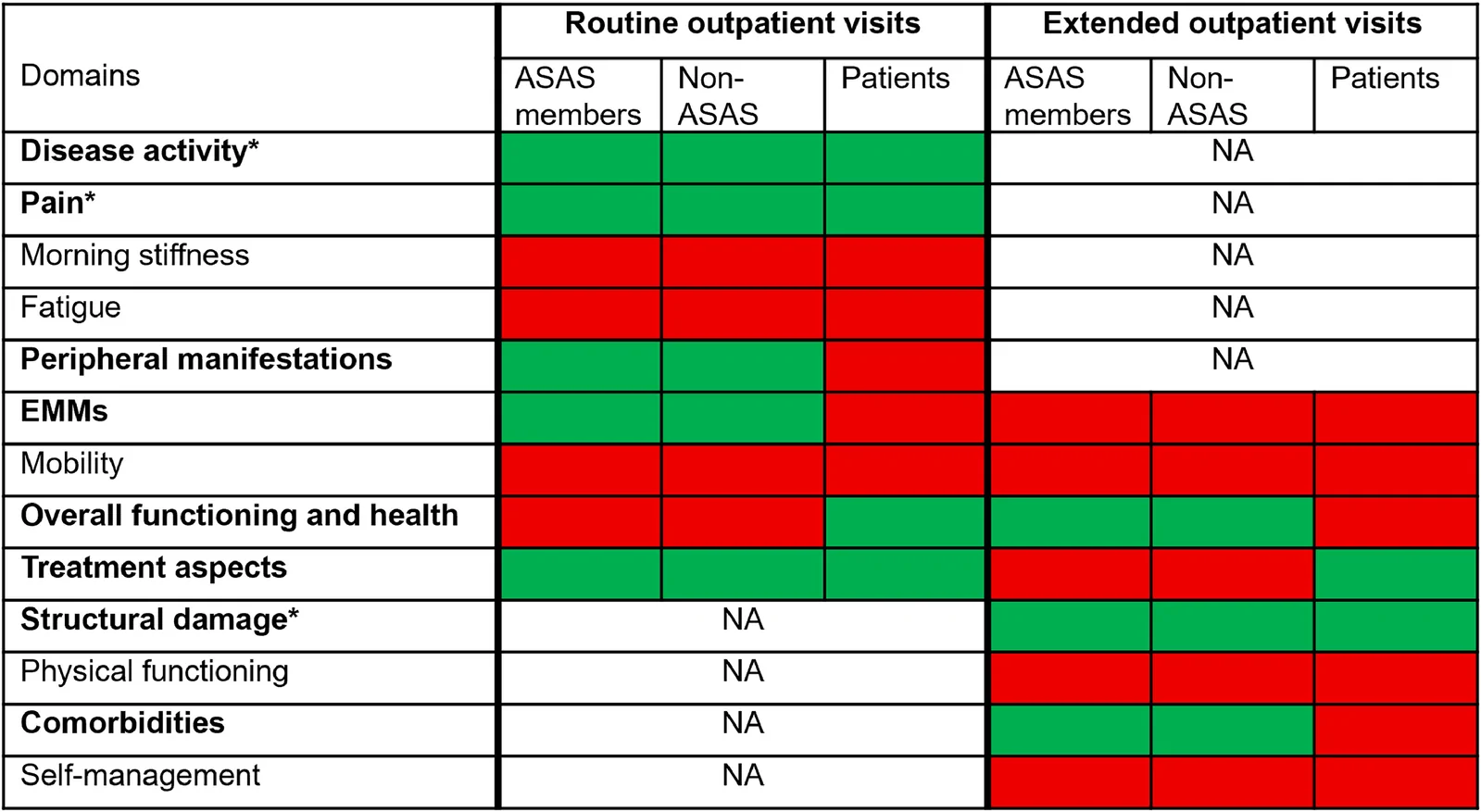
Candidate domains considered clinically relevant from the perspectives of stakeholders. All domains included in the second Delphi round are shown, and the selected domains are bolded. For each stakeholder group, domains that fulfilled the selection criteria within that group are coloured green, and domains that did not are coloured red. Domains that were preselected following round 1 are indicated with an asterisk (*), based on the criteria of being among the 5 highest-ranked domains by weighted scoring and rated as relevant by ≥70% in all stakeholder groups. The other bolded domains were selected following round 2, based on the criteria of being among the 5 highest-ranked domains and included in the rankings of >50% in any stakeholder group (based on combined data for routine and extended visits, as domains that were available for voting for both routine and extended visits could only be included by participants in their ranking for 1 of the 2 visit types). ASAS, Assessment of SpondyloArthritis international Society; EMM, extra-musculoskeletal manifestation; NA, not available as option; non-ASAS, non-ASAS member rheumatologists.

### Selection of instruments

The candidate instruments included in the third Delphi round for each of the 8 selected domains are presented in Table 1. No additional candidate instruments were adopted based on participant suggestions. Although patients did not participate in instrument selection through the survey, patient representatives contributed to the interpretation of results.

**Table 1:** Candidate instruments to be considered for the ASAS core set for the assessment of axial spondyloarthritis in clinical practice

*Disease activity*
• ASDAS • BASDAI • Patient global assessment of disease activity on a 0-10 NRS • CRP • Qualitative assessment of MRI of the SIJ for inflammatory lesions • Qualitative assessment of MRI of the spine for inflammatory lesions
*Pain*
• Total back pain on an NRS (BASDAI question 2/ASDAS question 1): ‘How would you describe the overall level of axSpA neck, back, or hip pain you have had?’
*Peripheral manifestations*
Peripheral arthritis • Targeted history taking, eg, ‘Do you have arthritis, or have you had arthritis since the last visit?’ and physical examination of the affected joints • Targeted history taking, and SJC44 if this indicates arthritis • Targeted history taking, and DAPSA44 score if this indicates arthritis • SJC44 in all situations • DAPSA44 score in all situations • Joint pain and swelling on an NRS (BASDAI question 3/ASDAS question 4): ‘How would you describe the overall level of pain/swelling in joints other than the neck, back, or hips you have had?’ Enthesitis • Targeted history taking, eg, ‘Do you have enthesitis, or have you had enthesitis since the last visit?’ and physical examination of the affected entheses • Targeted history taking, and MASES if this indicates enthesitis • MASES in all situations • Tenderness on an NRS (BASDAI question 4): ‘How would you describe the overall level of discomfort you have had from any areas tender to touch or pressure?’ Dactylitis • Targeted history taking, eg, ‘Do you have dactylitis, or have you had dactylitis since the last visit?’ and physical examination of the affected digits • Targeted history taking, and full dactylitis count if this indicates dactylitis • Full dactylitis count in all situations
*Extra-musculoskeletal manifestations*
Uveitis • Targeted history taking, eg, ‘Have you had any signs or symptoms that are suggestive of uveitis since the last visit?’ • ASAS CRF for uveitis IBD • Targeted history taking, eg, ‘Have you had any signs or symptoms that are suggestive of IBD since the last visit?’ • ASAS CRF for IBD Psoriasis • Targeted history taking, eg, ‘Have you had any signs or symptoms that are suggestive of psoriasis (skin/nails) since the last visit?’ • ASAS CRF for psoriasis
*Treatment aspects*
Adherence • Targeted history taking, eg, ‘Have you taken your medications as prescribed and do you follow other interventions as advised?’ • Use of a specific instrument (eg, CQR) Safety • Targeted history taking, eg, ‘Have you experienced any side effects of your medication since the last visit?’
*Structural damage*
• Radiography of the SIJ • Radiography of the spine • CT of the SIJ • CT of the spine • MRI of the SIJ • MRI of the spine
*Overall functioning and health*
• ASAS Health Index
*Comorbidities*
• Targeted history taking, eg, ‘Have you been diagnosed with a new health condition since the last visit? If yes, have any new treatments been initiated?’ • Documentation of risk factors and screenings as recommended by EULAR • Documentation of vaccinations as recommended by EULAR • Use of a specific instrument (Charlson, mSCQ, RDCI, FCI, Elixhauser, or EULAR standardised reporting form)
*Mobility*
• Chest expansion • Occiput-to-wall distance • Tragus-to-wall distance • Cervical rotation • Lateral spinal flexion • Modified Schober test • Intermalleolar distance • BASMI

ASAS, Assessment of SpondyloArthritis international Society; ASDAS, Axial Spondyloarthritis Disease Activity Score; axSpA, axial spondyloarthritis; BASDAI, Bath Ankylosing Spondylitis Disease Activity Index; BASMI, Bath Ankylosing Spondylitis Metrology Index; CQR, Compliance Questionnaire for Rheumatology; CRF, case report form; CRP, C-reactive protein; CT, computed tomography; DAPSA44, Disease Activity Index for Psoriatic Arthritis based on 44 joints; EULAR, European Alliance of Associations for Rheumatology; FCI, Functional Comorbidity Index; IBD, inflammatory bowel disease; MASES, Maastricht Ankylosing Spondylitis Enthesitis Score; MRI, magnetic resonance imaging; mSCQ, Modified Self-administered Comorbidity Questionnaire; NRS, numerical rating scale; RDCI, Rheumatic Disease Comorbidities Index; SIJ, sacroiliac joints; SJC44, 44-swollen joint count.

Based on the prespecified selection criteria, 1 recommended instrument was derived for each of the 5 routine visit domains (Fig 4). A recommended instrument was selected for only 1 extended visit domain (‘comorbidities’). In addition, several optional instruments were selected across the domains. Notably, no instrument met the criteria for selection as either recommended or optional for ‘overall functioning and health’.

**Figure 4:**
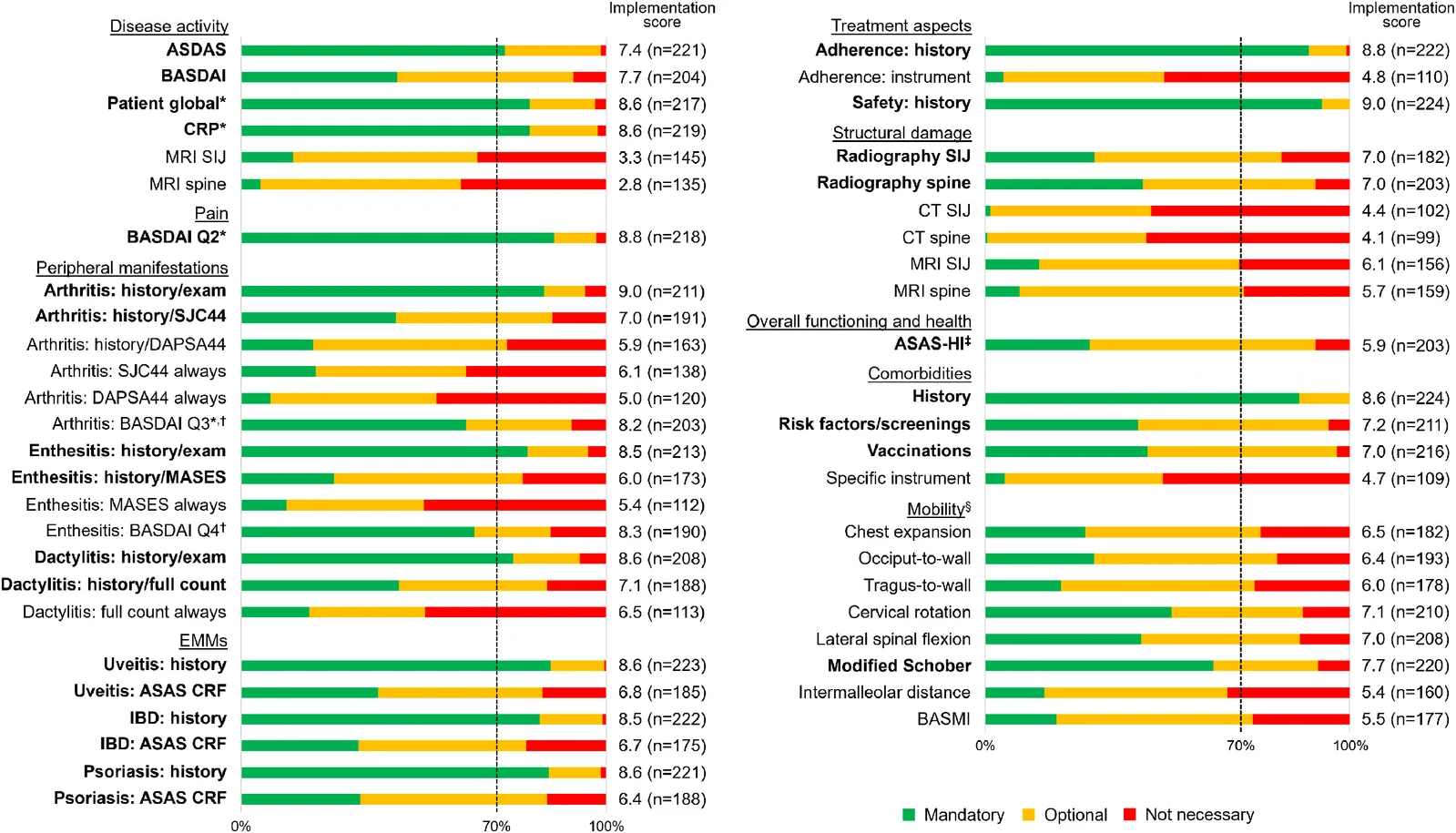
Clinical relevance and implementation scores of the candidate instruments for the selected domains from the perspectives of stakeholders. Combined data from ASAS members and non-ASAS rheumatologists are shown (n = 241 for ‘mobility’, n = 224 for all other domains). The bars indicate the percentage of participants who rated each instrument as ‘mandatory’, ‘optional’, or ‘not necessary’, with the 70% cut-off denoted by the dashed line. Participants who rated an instrument as ‘mandatory’ or ‘optional’ also provided a likelihood of implementation score for that instrument in clinical practice (0-10); the mean scores and corresponding number of participants (n) providing a score for each instrument are indicated next to the bars. Instruments selected as either recommended (rated as ‘mandatory’ by ≥70% and mean implementation score ≥7) or optional (rated as ‘mandatory’ or ‘optional’ by ≥70% and mean implementation score ≥6) are bolded. *Patient global assessment of disease activity, CRP, and BASDAI questions 2 and 3 are components of the ASDAS. ^†^Despite meeting the selection criteria for inclusion as an optional instrument, the working group excluded BASDAI questions 3 and 4 due to limited validity, in favour of the other selected instruments. ^‡^Despite marginally not satisfying the inclusion threshold as an optional instrument, the working group selected the ASAS-HI considering the high importance of the ‘overall functioning and health’ domain to patients, and to ensure all domains had at least 1 corresponding instrument. ^§^Six instruments met the threshold for inclusion as an optional instrument for ‘mobility’. To uphold the feasibility of the core set, the steering committee tentatively selected the highest rated of these 6, the modified Schober test. ASAS, Assessment of SpondyloArthritis international Society; ASAS-HI, ASAS Health Index; ASDAS, Axial Spondyloarthritis Disease Activity Score; BASDAI, Bath Ankylosing Spondylitis Disease Activity Index; BASMI, Bath Ankylosing Spondylitis Metrology Index; CRF, case report form; CRP, C-reactive protein; CT, computed tomography; DAPSA44, Disease Activity Index for Psoriatic Arthritis based on 44 joints; EMM, extra-musculoskeletal manifestation; IBD, inflammatory bowel disease; MASES, Maastricht Ankylosing Spondylitis Enthesitis Score; MRI, magnetic resonance imaging; non-ASAS, non-ASAS member rheumatologists; SIJ, sacroiliac joints; SJC44, 44-swollen joint count.

Several reservations regarding this initial selection strategy were raised during subsequent working group discussions, prompting adaptations based on expert opinion and consultation with patient representatives. Specifically highlighted were the lack of objective instruments for (certain components of) the ‘peripheral manifestations’ and ‘EMMs’ domains, as well as the consequence that 1 domain would not have a corresponding instrument, leading to an incomplete coverage of the aspects considered important by stakeholders. To address these issues, the selection criteria for optional instruments were adjusted to be less stringent, reducing the required mean implementation score to ≥6. This resulted in the addition of 1 supplementary objective instrument for ‘peripheral manifestations’ and 3 for ‘EMMs’. In parallel, the working group opted to exclude the Bath Ankylosing Spondylitis Disease Activity Index questions 3 and 4 for ‘peripheral manifestations’ due to their limited validity in accurately capturing peripheral arthritis and enthesitis, respectively, in favour of the (newly) selected quantitative instruments. Although no instrument for ‘overall functioning and health’ formally met the adjusted criteria, the working group nonetheless selected the ASAS Health Index, given the high importance of the domain to patients and considering that this instrument only marginally did not satisfy the inclusion threshold.

### Additional (fourth) Delphi round

Following evaluation of the selected domains and instruments, the absence of a domain pertaining to the physical examination of axial manifestations of axSpA was raised as a point of concern within the working group, informed by expert opinion emphasising the importance of this particular aspect. A fourth Delphi round was therefore undertaken, with the aim of re-evaluating the earlier exclusion of the ‘mobility’ domain. Participants were asked to indicate if this domain should be included, and ASAS members and non-ASAS rheumatologists also appraised candidate instruments in the same manner as in the third Delphi round (Supplementary File S2).

A response was received from 163 of 201 (81%) ASAS members, 78 of 173 (45%) non-ASAS rheumatologists, and 71 of 167 (43%) patients. The majority of participants indicated a preference to include ‘mobility’ (ASAS members and non-ASAS rheumatologists: 53%, patients: 68%), prompting its addition as a supplementary domain for extended visits, in accordance with the same selection criteria applied for domain selection in the second round. Six instruments met the criteria for inclusion as an optional instrument to assess ‘mobility’ (Fig 4). To uphold the feasibility of the core set, the steering committee tentatively selected the highest rated of these 6, the modified Schober test.

## DISCUSSION

The ASAS-PRAXIS project aims to develop a core set for the assessment of axSpA in clinical practice, thereby contributing towards improved standardisation of disease follow-up. The present study represents the first step by identifying clinically relevant domains and corresponding instruments. Based on stakeholder preferences, the working group selected 5 domains for routine outpatient visits and 4 supplementary domains for extended outpatient visits as the basis for developing the core set. One recommended instrument was selected for each routine visit domain, alongside several optional instruments. At least 1 recommended or optional instrument was selected for each extended visit domain.

The selected domains capture numerous facets of the disease burden of axSpA, including disease manifestations and progression, associated health factors, function, and treatment aspects. This selection therefore facilitates a comprehensive assessment of both the disease process itself and its consequences, resulting in a holistic representation of the disease at the individual level of each patient. Systematic collection of this information would support well-informed decision-making regarding pharmacological and nonpharmacological treatment. Simultaneously, the constrained number of selected domains—afforded by the ranking exercises which forced participants to prioritise their choices—helps to balance multidimensionality with practicality of assessments. The extent to which this approach proves feasible in clinical practice, however, remains to be formally evaluated following finalisation of the core set. Feasibility may vary between healthcare systems depending on existing care delivery structures and available resources, with implementation in low-resource settings likely requiring particular attention.

All stakeholder groups agreed on the domains with the highest priority, namely ‘disease activity’, ‘pain’, and ‘structural damage’. This is, remarkably, largely in alignment with the ASAS-EULAR management recommendations for axSpA, which emphasise the importance of assessing and intervening on disease activity, function, and structural damage in particular [1]. Although ASAS members and non-ASAS rheumatologists indicated similar preferences regarding the remaining domains, patients expressed different priorities. HCPs tended to rank domains pertaining to objective disease manifestations higher, whereas patients generally prioritised patient-experienced aspects and identified a larger number of domains as important. This voting pattern was also observed in the earlier Delphi survey informing the update of the ASAS COS for research settings [13]. Ultimately, however, higher concordance emerged across all groups as the Delphi survey progressed, resulting in a selection that is grounded in the collective priorities of all stakeholder groups. Although certain domains initially prioritised by patients were not retained, possibly reducing the focus on patient-experienced aspects during follow-up, the selected domains nevertheless reflect patient input and do not preclude the assessment of additional domains if deemed necessary. The robustness of the selection process was further strengthened through periodic critical discussions within the working group, during which expert opinion was structurally incorporated. These discussions culminated, for example, in a fourth Delphi round to validate stakeholder opinions on the domain selection, ensuring all decisions were well-substantiated.

With respect to instruments, ASAS members and non-ASAS rheumatologists again made largely similar choices. As with the domains, the selected instruments are therefore representative of the preferences of diverse stakeholders. Offering both recommended and optional instruments provides further flexibility to HCPs to implement the eventual core set in a manner best suited to their preferences and local context. A noteworthy observation was the widespread preference among HCPs to use history taking to assess several domains, as opposed to a specific measurement instrument. Plausibly, history taking was viewed as the most feasible and accessible option as it aligns with the established clinical routines of HCPs and requires no additional training.

Several limitations should be acknowledged. First, attrition of participants led to progressively smaller sample sizes, which is a known phenomenon in Delphi surveys [16]. This may have influenced the level of consensus through attrition bias. However, only minor differences were observed between the characteristics of responders and nonresponders to subsequent rounds, indicating that attrition was largely random and unlikely to have had a substantial impact. Measures to mitigate attrition included personalised invitations to patients, reminders, and retaining participants in all rounds, even if an earlier round was missed. Second, the geographical distribution of participants was somewhat disproportionate. Although representation from all continents was achieved, over half of the participating ASAS members and non-ASAS rheumatologists originated from Europe, which is attributable to the composition of the ASAS membership. The distribution of patients was also restricted by the requirement of proficiency in English or Spanish. These demographic factors possibly contributed to some extent of underrepresentation of certain perspectives. Lastly, the preferences of participants might have been subject to confirmation bias. ASAS members, many of whom contributed to the development of previous outcome frameworks, may have been inclined to vote in alignment with established assessment approaches. Likewise, non-ASAS rheumatologists, who by design did not have a specific clinical or research focus on axSpA, could have favoured domains and instruments based on current practices and familiarity rather than clinical validity, due to comparatively lower exposure to the available options. Nevertheless, the progressive convergence of preferences across all stakeholder groups suggests that any resulting influence on the selection process was minimal. Notwithstanding these limitations, this study resulted in a comprehensive selection of domains and instruments that reflects the perspectives of a substantial number of HCPs and patients across diverse healthcare settings worldwide. Once finalised, this selection is expected to translate into a core set with widespread applicability.

In conclusion, this first step of the ASAS-PRAXIS project identified domains and corresponding instruments considered relevant by stakeholders, which will facilitate ASAS in developing the core set for the assessment of axSpA in clinical practice. Subsequent steps include evaluating the feasibility of the selected domains and instruments and discussing them within the ASAS community at the ASAS annual meeting in January 2026 to achieve consensus and endorse the core set.

## Competing interests

MLS has received honoraria from Novartis. VN-C has received research grants, honoraria, and consultancy fees from AbbVie, Alfasigma, BMS, Fresenius Kabi, Galapagos, Janssen, Johnson & Johnson, Eli Lilly, MoonLake, MSD, Novartis, Pfizer, Roche, and UCB; and is the ASAS president and a member of the ASAS Executive Committee. XB has received research grants from AbbVie, Celltrion, Janssen, MoonLake, and Novartis; honoraria and consultancy fees from AbbVie, Advanz, Alexion, Alfasigma, Amgen, AstraZeneca, BMS, CESAS, Celltrion, Clarivate, Dr. Reddy’s, Eli Lilly, Galapagos, Greywolf, GlobeMed, Johnson & Johnson, MoonLake, Novartis, Peervoice, Pfizer, Roche, Sandoz, Springer, STADA, Takeda, UCB, and Zuellig; and is the EULAR president, ASAS immediate past-president, and a member of the ASAS Executive Committee. AM has received research grants from AbbVie, Biogen, and UCB; and honoraria and consultancy fees from, and participated in advisory boards for, AbbVie, Alfasigma, Eli Lilly, Johnson & Johnson, Novartis, Pfizer, and UCB. EN has received research grants from Eli Lilly, Pfizer, and UCB; and honoraria from, and participated in advisory boards for, AbbVie, Alfasigma, Eli Lilly, Fresenius Kabi, Galapagos, Gilead, Novartis, Pfizer, and UCB. WB-M has received honoraria and consultancy fees from AbbVie, Amgen, Fresenius Kabi, Janssen, Johnson & Johnson, Novartis, and UCB-Biopas; and is a member of the ASAS Executive Committee. PC has received consultancy, advisory, and speaker fees/honoraria for lecturing and attendance at scientific exchange meetings from AbbVie, Eli Lilly, Janssen, and UCB; and research funding from MSD, Pfizer, and UCB. AC declares no competing interests. JD declares no competing interests. WF has received speaker honoraria from DKSH, GSK, Johnson & Johnson, and Novartis; consultancy fees from AbbVie, Eli Lilly, Johnson & Johnson, and Novartis; conference sponsorships from Abbott, AbbVie, Boehringer Ingelheim, DKSH, Johnson & Johnson, Novartis, and Roche; and research grants from DKSH, Johnson & Johnson, and Novartis. RG-S has received research, speaker, and advisory grants from AbbVie, Adium, BMS, Eli Lilly, Johnson & Johnson, Novartis, and Pfizer. LG has received research grants from AbbVie, Eli Lilly, Novartis, and UCB; consulting fees from AbbVie, Alfasigma, Amgen, BMS, Celltrion, Eli Lilly, Johnson & Johnson, MoonLake, Novartis, Nordic Pharma, Oruka, Pfizer, STADA, Takeda, and UCB; and nonfinancial support from AbbVie, Amgen, Biogen, BMS, Celltrion, Eli Lilly, Johnson & Johnson, Pfizer, and UCB. DvdH has received consulting fees from Alfasigma, BMS, Greywolf, Janssen, Takeda, and UCB; holds stocks of Altis Medicines Inc.; and is an associate editor of Annals of the Rheumatic Diseases and director of Imaging Rheumatology BV. DK has received grants, honoraria, and speaker fees from AbbVie, Alfasigma, AstraZeneca, BMS, Boehringer Ingelheim, Celltrion, Eli Lilly, Galapagos, GSK, Janssen, Merck, MSD, Novartis, Roche/Chugai, Sanofi, and UCB. UK has received grant and research support and consultancy fees from AbbVie, Alfasigma, Amgen, BIOCAD, Biogen, BMS, Chugai, Eli Lilly, Fresenius Kabi, Gilead, GBA, Grünenthal, GSK, Hexal, Johnson & Johnson, MSD, Novartis, onkowissen.de, Pfizer, Roche, UCB, and Viatris; and is a member of the ASAS Executive Committee. YHK declares no competing interests. JL declares no competing interests. MM has received consulting fees from AbbVie, BMS, Eli Lilly, Kyowa Kirin, Johnson & Johnson, Novartis, Pfizer, Priovant, and UCB. DP has received research support from AbbVie, BMS, Eli Lilly, Johnson & Johnson, Novartis, Pfizer, and UCB; consulting fees from AbbVie, Eli Lilly, GSK, Greywolf, Johnson & Johnson, Merck, MoonLake, Novartis, Pfizer, and UCB; speaker fees from AbbVie, Canon, Celltrion, Eli Lilly, GlobeMed, Johnson & Johnson, Medscape, Novartis, Peervoice, Pfizer, and UCB; and is a member of the ASAS Executive Committee. SR has received research grants from AbbVie, Alfasigma, Eli Lilly, MSD, Novartis, Pfizer, and UCB; consultancy fees from AbbVie, Alfasigma, Eli Lilly, Johnson & Johnson, MSD, Novartis, Pfizer, Takeda, and UCB; and is a member of the ASAS Executive Committee and EULAR Quality of Care Committee. AS has received research grants from AbbVie and speaking and consulting fees from AbbVie, Eli Lilly, Janssen, and MSD. NZ has received speaker fees from AbbVie, Apotex, Eli Lilly, Janssen, Novartis, Pfizer, Pharmaline, Pierre Fabre, and Roche; research grants from AbbVie, Algorithm/Celgene, NewBridge, and Pfizer; and participated in advisory boards for AbbVie, Eli Lilly, Gilead, Janssen, NewBridge, Novartis, Pfizer, and Roche. AvT has received research grants from AbbVie, Novartis, and UCB; honoraria from Novartis and Pfizer; and consultancy fees from AbbVie, Johnson & Johnson, Novartis, and UCB.

## Appendix Supplementary materials

Image, application 1

## Footnotes

Supplementary material associated with this article can be found in the online version at doi:10.1016/j.ero.2026.100246.
