# Supplementary material for: Selection of relevant domains and instruments for the ASAS core set to assess axial spondyloarthritis in clinical practice: a Delphi survey of stakeholders
Source: EULAR Rheumatol Open. 2026 Oct 7;2(4):100246. doi: 10.1016/j.ero.2026.100246 (PMC13676701; doi:10.1016/j.ero.2026.100246)
Supplement: Supplementary file 1 [file mmc1.docx]

**Supplementary material for “Selection of relevant domains and instruments for the ASAS core set to assess axial spondyloarthritis in clinical practice: a Delphi survey of stakeholders”**

**Supplementary File S1.** Search strategy of the scoping literature review to identify potential domains to assess in axial spondyloarthritis and other rheumatic and musculoskeletal diseases in clinical practice

((((((((((((((((rheumat*[Title/Abstract]) OR reumat*[Title/Abstract]) OR arthrit*[Title/Abstract]) OR artrit*[Title/Abstract]) OR "arthritis, rheumatoid"[MeSH Terms]) OR psoriatic*[Title/Abstract]) OR arthritis, psoriatic[MeSH Terms]) OR spondylitis*[Title/Abstract]) OR spondyloarthropat*[Title/Abstract]) OR spondyloarthritis[Title/Abstract]) OR spondylitis, ankylosing[MeSH Terms]) OR ("Axial Spondyloarthritis"[Mesh])) AND (((((((((((("Guideline" [Publication Type]) OR "Guidelines as Topic"[Mesh]) OR "Practice Guideline" [Publication Type]) OR "Consensus"[Mesh]) OR "Standard of Care"[Mesh]) OR (guideline)) OR (recommendation)) OR (consensus)) OR ("standard of care")) OR ("standards of care"))) OR (reference))) AND (((((((("Clinical Medicine"[Mesh]) OR (clinical practice)) OR (daily practice)) OR (clinic)) OR (outpatient)) OR (functioning)) OR (everyday)) OR (routine care))) AND (((((((("Patient Outcome Assessment"[Mesh]) OR "Outcome Assessment, Health Care"[Mesh]) OR "Treatment Outcome"[Mesh])) OR (outcome)) OR (assessment)) OR (evaluation)))) AND ((((((((domain) OR (category)) OR (concept)) OR (aspect)) OR (instrument)) OR (frequency)) OR (setting)) OR (scenario))) AND ((((domain) OR (category)) OR (concept)) OR (aspect))

**Supplementary File S2.** Elaboration on the format of the Delphi rounds

**First Delphi round**

The first Delphi round aimed to evaluate the clinical relevance of the candidate domains identified in the literature review. Participants were first asked to indicate whether they considered each of the candidate domains relevant to assess during routine or extended visits, with the option to also suggest new domains. Participants then ranked up to five domains they deemed most relevant during routine visits, and up to an additional five domains for extended visits. A weighted scoring system was applied, in which domains were assigned point values according to their rank per participant, with total scores summed across each stakeholder group to generate a prioritised list. For illustration, 1 point was assigned each time a domain was ranked first, 0.8 points when ranked second, 0.6 when ranked third, 0.4 when ranked fourth, and 0.2 when ranked fifth. For each visit type, domains that fulfilled the selection criteria in all stakeholder groups were considered pre-selected, and those that fulfilled the criteria in at least one stakeholder group were offered for re-ranking in a second round of voting.

Left: example of the exercise regarding the relevance of domains, Right: example of the ranking exercise.

**Second Delphi round**

The second Delphi round aimed to further refine and prioritise the selection of candidate domains. Domains carried forward from the first round were presented to participants for re-ranking. Up to five domains (with pre-selected domains from the first round counting toward the total) could again be ranked for routine visits, and up to an additional five for extended visits. Importantly, if a domain was available for voting for both routine and extended visits, participants could only include it in their ranking for one of the two visit types.

Left: example of the re-ranking exercise for routine visits, Right: example of the re-ranking exercise for extended visits.

**Third Delphi round**

The third Delphi round aimed to select instruments to assess the chosen domains. Participants were first asked to categorise each candidate instrument as ‘mandatory’, ‘optional’ or ‘not necessary’ for clinical practice. Next, for each instrument rated as ‘mandatory’ or ‘optional’, participants indicated their likelihood of implementing it in clinical practice on a 0-10 numerical rating scale. Alternative instruments could also be suggested.

Left: example of the exercise regarding the relevance of instruments, Right: example of the questions on the likelihood of implementing instruments.

**Additional (fourth) Delphi round**

A fourth Delphi round was conducted following working group discussions to re-evaluate the exclusion of the ‘mobility’ domain. Participants were asked to indicate if this domain should be included and appraise candidate instruments in the same manner as in the third round.

Left (above): example of the question evaluating the exclusion of ‘mobility’, Right: example of the exercise regarding the relevance of instruments, Left (below): example of the questions on the likelihood of implementing instruments.

**Supplementary Figure S1.** Flowchart of the inclusion of articles in the scoping literature review to identify potential domains to assess in axial spondyloarthritis and other rheumatic and musculoskeletal diseases in clinical practice.

**Supplementary File S3.** Summary of the results from the scoping literature review to identify potential domains to assess in axial spondyloarthritis and other rheumatic and musculoskeletal diseases in clinical practice

Elaboration on data extraction:

- If a specific instrument was reported as relevant, the domain to which it pertains was extrapolated. For example, ‘C-reactive protein’ and ‘Patient/physician global assessment of disease activity’ were extrapolated to ‘Disease activity’.
- In studies where agreeability to a pre-determined set of domains was investigated, only domains that were concluded to be relevant were extracted (e.g. based on an agreement of >50% of participants).
- In studies where the International Classification of Functioning, Disability and Health (ICF) model was utilised to categorise and present relevant aspects in disease management, overarching domains were extrapolated from the reported ICF codes.

| **Ref.** | **Year** | **Diseases covered** | **Stakeholders involved in study*** | **Domains highlighted** | **Frequency of assessment** | **Specific scenarios for assessment** |
| --- | --- | --- | --- | --- | --- | --- |
| ***Articles with relevance to axSpA*** | | | | | | |
| (1) | 2024 | axSpA | HCPs | Disease assessment; Comorbidities; Lifestyle; QoL | All domains: every year | NR |
| (2) | 2023 | All RMDs | NA | Pain; Change in appearance; Activity limitations; Social isolation; Work disruption; Financial burden; Emotional impact | NR | NR |
| (3) | 2022 | axSpA | HCPs | Morning stiffness; EAMs; Medication use; Axial pain; Disease activity | NR | NR |
| (4) | 2022 | SpA | HCPs | ^a^ Disease activity  ^b^ (Residual) pain; Musculoskeletal manifestations and EAMs; Patient expectations and concerns; Comorbidities; Lifestyle; Treatment compliance and tolerance (DMARDs); Information sources; Structural damage | ^a^ Specifically 3 and 6 months after starting new therapy  ^b^ NR | All domains: choice of domains should be adapted to the individual |
| (5) | 2021 | axSpA | HCPs | Disease activity; Musculoskeletal manifestations and EAMs; Structural damage; Adverse effects of treatment; Vaccination status; Comorbidities | All domains: frequency adapted to symptoms, severity, and treatment | NR |
| (6)^†^ | 2021 | axSpA | HCPs and patients | ^a^ Disease activity; Pain; Morning stiffness; Fatigue; Physical function; Overall functioning and health; Adverse events  ^b^ EMMs; Peripheral manifestations; Structural damage  ^c^ Spinal mobility; Sleep; Work and employment | NR | ^a^ All trials  ^b^ DMARD trials only  ^c^ Optional |
| (7) | 2020 | axSpA | HCPs | ^a^ Nocturnal pain; Morning stiffness  ^b^ Disease activity; Extra-rheumatological manifestations; Comorbidities  ^c^ Mobility | ^a^ Every visit  ^b^ Every 6-12 months  ^c^ Every 1-2 years | NR |
| (8) | 2020 | axSpA | HCPs and patients | ^a^ Disease activity (symptoms, severity)  ^b^ Comorbidities; Employment; Psychological factors; Lifestyle and physical activity | ^a^ Every 6 months  ^b^ Every year | All domains: choice of domains should be adapted to the individual |
| (9) | 2019 | axSpA, JIA, PsA, RA | HCPs and patients | ^a^ Serious adverse effects  ^b^ Disease activity  ^c^ Pain; Fatigue; Activity limitations; Overall physical and mental health impact; Work, school, household ability, and productivity; Treatment response  ^d^ Demographics; Smoking status; Comorbidities; Disease characteristics (time since diagnosis, seropositivity) | ^a^ Every visit  ^b^ At least every 6 months in an HDA state, every year in an ID/LDA state, at discretion of patient and HCP  ^c^ Every year  ^d^ At first assessment | See frequency |
| (10) | 2018 | axSpA | Patients | Oral health | NR | NR |
| (11) | 2018 | SpA | HCPs and patients | Musculoskeletal disease activity; Cutaneous manifestations and EAMs; Comorbidities; Patient factors; Drug-related risks | All domains: every 1-3 months in an HDA state, every 6-12 months in an ID/LDA state | See frequency |
| (12) | 2017 | SpA | NA | Physical aspects (e.g. pain, fatigue, sleep); Psychological aspects (e.g. positive/negative feelings, cognition, self-esteem, bodily image and appearance); Level of independence (e.g. mobility, ADL, medication dependence, work capacity); Social relationships (e.g. personal relationships, social support, sexual activity); Environment (e.g. freedom, physical safety, security, home environment, work satisfaction, financial resources, health and social care, opportunities for learning, participation in leisure activities, physical environment, transport); Personal beliefs (spirituality, religion); QoL and general health perception (e.g. treatment burden) | NR | NR |
| (13) | 2017 | All RMDs | HCPs | Conceptional aspects (e.g. obstetric-gynaecological history, pregnancy contraindications, maternal-foetal risk factors, contraception); Disease activity and status; Treatment status; Comorbidities | NR | All domains: patients of reproductive age |
| (14) | 2016 | All RMDs | HCPs and patients | Comorbidities | Every 5 years | NR |
| (15) | 2016 | All RMDs | HCPs | Comorbidities | Variable per comorbidity, range of every 3 months-20 years, or on indication | Depth of assessment depends on the individual |
| (16) | 2016 | axSpA | HCPs | ^a^ Musculoskeletal factors (e.g. axial mobility, chest expansion); Psychosocial factors; Physical activity and adherence  ^b^ Safety (e.g. strength, balance, mobility changes, postural stability, cardiorespiratory function); Comorbidities; Structural damage | All domains: every year, frequency adapted to symptoms, function, and mobility | ^a^ All patients  ^b^ Adapted to the individual (e.g. disease stage) |
| (17) | 2009 | axSpA | Patients | Mental functions; Functions of body systems and organs; Pain, Movement and mobility; Cognition; Self-care and ADL; Support and relationships; Employment; Recreation and leisure; Use of products and technology; Attitudes to disease; Use of services, systems, and policies | NR | NR |
| (18) | 2009 | axSpA | HCPs | Mental functions; Pain; Functions of body systems and organs; Movement and mobility; Self-care and ADL; Employment; Recreation and leisure; Use of products and technology; Support and relationships; Attitudes to disease; Use of services, systems, and policies | NR | NR |
| (19) | 2008 | axSpA | HCPs | Disease activity and severity; (Nocturnal) pain; Daily function; EAMs; Peripheral arthritis; Structural damage; Mobility | NR | NR |
| ***Articles with relevance to other RMDs*** | | | | | | |
| (20) | 2024 | SLE | HCPs and patients | ^a^ Depression; Physical function  ^b^ Cognition; Fatigue | ^a^ Every year  ^b^ Optional | NR |
| (21) | 2024 | RA | HCPs and patients | ^a^ Disease activity  ^b^ Comorbidities and risk factors  ^c^ Structural damage; EAMs; Medical history (e.g. surgery); Treatment adherence and tolerance; Patient’s opinion and feelings; Pregnancy; Travel; Vaccination status; Oral health | ^a^ Every 1-3 months in active disease  ^b^ Every 5 years (specifically CVRM)  ^c^ NR | See frequency |
| (22) | 2023 | RA | Patients | Pain; Disease activity; Stiffness; Physical function; Fatigue; Emotional distress; Participation; Sleep | NR | NR |
| (23) | 2023 | RA | HCPs and patients | ^a^ Disease activity  ^b^ Comorbidities and risk factors  ^c^ Safety issues; Structural damage | ^a^ Every 1-3 months in active disease  ^b^ NR  ^c^ NR | ^a^ See frequency  ^b^ Specifically CVRM and malignancies with JAKi use  ^c^ NR |
| (24) | 2022 | PsA | HCPs and patients | Disease activity; Peripheral manifestations; Axial disease; EMMs; Pain; Function; QoL; Structural damage; Comorbidities; Reproductive health, Patient factors (e.g. cost, preferences) | “Clinical response” every 3-6 months after starting new therapy | See frequency |
| (25) | 2021 | Fibromyalgia | Patients | Work and school | NR | NR |
| (26) | 2019 | Myositis | HCPs, patients, and informal caregivers | Symptoms of organ involvement (muscles, skin, lungs, gastrointestinal, joints); Fatigue; Physical activity; Medication side effects; Pain; Infections; Work ability; Cognitive impact; Emotional distress; Interaction with HCPs/authorities | NR | NR |
| (27) | 2018 | PsA | HCPs | Disease activity; Peripheral manifestations; Axial manifestations; Pain; Fatigue; Overall assessment of the patient; Functional capacity; QoL; Comorbidities | “Clinical activity”: every month in an HDA state, every 3 months in an ID/LDA state | See frequency |
| (28) | 2017 | RA | HCPs | Disease activity; Physical function; QoL | NR | NR |
| (29) | 2017 | OA | Patients | Pain; Stiffness; Disease activity; ADL and leisure; Psychological wellbeing; Aesthetic changes; Social function and participation; Work productivity | NR | NR |
| (30) | 2017 | RA | Patients | Physical function; Pain; ADL; Work; Fatigue; Social function; Emotional function | NR | NR |
| (31) | 2017 | PsA | HCPs | Disease activity; EMMs; Peripheral manifestations; Axial manifestations; Structural damage; Physical function; QoL; Fatigue; Functional impairment; Comorbidities; Treatment risks and safety | “Therapeutic targets”: every 3 months in active disease, every 6-12 months in stable disease; safety at every visit | See frequency |
| (32) | 2017 | SjD | Patients | Pain; Dryness; Physical complaints; Treatment side effects; Psychological and emotional challenges; Social life and daily living | NR | NR |
| (33) | 2016 | Vasculitis | HCPs and patients | ^a^ Disease activity and status; Organ involvement and complications; Treatment safety and side effects  ^b^ Comorbidities and impact | ^a^ Every 1-3 months  ^b^ NR | All domains: depth of assessment depends on the individual |
| (34) | 2016 | RA | HCPs and patients | ^a^ Disease activity  ^b^ Comorbidities; Patient factors; Drug-related risks; Structural changes; Functional impairment and work performance | ^a^ Every month in a high/moderate disease activity state, every 6 months for LDA/remission  ^b^ NR, to be considered when making clinical decisions | See frequency |
| (35) | 2015 | Myositis | Patients | Symptoms (muscles, lungs, skin); Pain; Stiffness and discomfort, Fatigue, Insomnia, Cognitive function; ADL and participation; Strategies and recovery; Disease knowledge and self-management; Emotional factors | NR | NR |
| (36) | 2015 | PMR | HCPs and patients | Disease activity; Comorbidities; Treatment risk factors and side effects; Medication use; Risk factors for relapse/prolonged therapy | All domains: every 1-2 months in first year, every 2-3 months in second year, subsequently on indication | NR |
| (37) | 2015 | Gout | HCPs | ^a^ Treatment target  ^b^ Medication safety  ^c^ Comorbidities; Risk factors | ^a^ Every 3-6 months  ^b^ Variable depending on medication type  ^c^ NR | See frequency |
| (38) | 2015 | RA | Patients | Patient’s attitude and adaptability; Coping and resilience; Diet and weight; Patient involvement and self-efficacy; Job satisfaction; Individual/societal activities; Coherence; Social appreciation | NR | NR |
| (39) | 2014 | RA | HCPs and patients | Pain; Function; Disease activity; Stiffness; Participation; Self-management; Fatigue | NR | NR |
| (40) | 2014 | Fibromyalgia, RA, SSc, SLE, PsA, OA | Patients | Attitudes to disease; Body image and appearance; Mental and emotional functions; Functions of body systems and organs; Disease activity and manifestations; Movement and mobility; Self-care and ADL; Paid work, productivity and school; Support and relationships; Recreation and leisure; Treatment and side effects, Diet; Use of products and technology; Use of services, systems, and policies; Information sources | NR | NR |
| (41) | 2013 | RA | Patients | Comorbidities | NR | NR |
| (42) | 2012 | RA | HCPs and patients | Pain; Function; Disease activity; Participation; Stiffness; Self-management; Fatigue | NR | NR |
| (43) | 2012 | RA | HCPs and patients | Disease activity; (Nocturnal) pain; Medication and healthcare use/dependence; Fatigue; Emotional function; Morning stiffness; EAMs | NR | NR |
| (44) | 2010 | RA | Patients | QoL; ADL; Physical function; Work; Pain; Disease activity; Fatigue; Deformities; Disease knowledge; Social support; Self-management | NR | NR |
| (45) | 2010 | SLE | HCPs | ^a^ Comorbidities and risk factors (especially CVRM); Physical activity; Vaccination status; Medication use and drug toxicity; Disease activity; QoL; Organ/mucocutaneous involvement; Neuropsychiatric manifestations; Occupational problems; Pregnancy planning  ^b^ Damage | ^a^ Every 6-12 months in inactive disease, adapted to disease activity, damage, comorbidities, and medication use  ^b^ Every year | See frequency |
| (46) | 2010 | RA | Patients | Pain; ADL; Structural damage; Mobility; Life enjoyment; Independence; Fatigue; Valued activities | NR | NR |
| (47) | 2010 | RA | HCPs | Disease activity; Morning stiffness; (Nocturnal) pain; Structural damage | Variable per rheumatologist | NR |
| (48) | 2009 | RA | HCPs | ^a^ Disease activity  ^b^ Functional impairment; Structural damage | ^a^ Every 3 months when not in remission, every 3-6 months in remission with rheumatologist or rheumatology nurse (with at least once a year with rheumatologist)  ^b^ Every year | See frequency |
| (49) | 2008 | Fibromyalgia | HCPs and patients | Pain; Treatment side effects; Mobility; Cognition; Energy/fatigue; Impact on daily living/health-related QoL; Emotional wellbeing; Sleep; Stiffness | NR | NR |
| (50) | 2007 | PsA | Patients | Body imaging and appearance; Sleep; Fatigue; Emotional function; Pain; Functions of body systems and organs; Skin and nails; Peripheral manifestations; Axial manifestations; Movement and mobility; Attitudes to disease; Recreation and leisure; Self-care and ADL; Paid work and productivity; Support and relationships; Treatment and side effects; Diet; Use of products and technology; Use of services, systems, and policies | NR | NR |
| (51) | 2005 | OA | HCPs | Risk factors; Physical activity; Comorbidities; Medication use; Pain; Disabilities; Structural damage; Patient wishes and expectations | NR | NR |

* Definition of this column varies by article type: for recommendations/consensus statements, this refers to the stakeholders consulted in the development process; for other study designs (other than systematic literature reviews), this refers to the stakeholders from whom data were collected.

^†^ Despite its focus on the research rather than clinical setting, the 2021 update of the ASAS-OMERACT core domain set for axSpA was included in this review as it formed the foundation of this project.

ADL: activities of daily living; ASAS: Assessment of SpondyloArthritis international Society; axSpA: axial spondyloarthritis; CVRM: cardiovascular risk management;
DMARD: disease-modifying anti-rheumatic drug; EAM: extra-articular manifestation; EMM: extra-musculoskeletal manifestation; HCP: healthcare professional; HDA: high disease activity; IBD: inflammatory bowel disease; ID: inactive disease; JAKi: Janus kinase inhibitor; JIA: juvenile idiopathic arthritis; LDA: low disease activity; NA: not applicable; NR: not reported; OA: osteoarthritis; OMERACT: Outcome Measures in Rheumatology; PMR: polymyalgia rheumatica; PsA: psoriatic arthritis; QoL: quality of life; RA: rheumatoid arthritis; RMD: rheumatic and musculoskeletal disease; SjD: Sjögren’s disease; SLE: systemic lupus erythematosus; SpA: spondyloarthritis; SSc: systemic sclerosis.

**References**

1.	Ruyssen-Witrand A, Caillet-Portillo D, Najm A, Fogel O, Baillet A, Claudepierre P, et al. Standardized reporting for systematic global evaluation of axial spondyloarthritis: An evidence-based and consensus-driven initiative. Joint Bone Spine. 2024;91(5):105733.

2.	Chapman LS, Flurey CA, Redmond AC, Richards P, Hofstetter C, Tapster B, et al. Living with foot and ankle disorders in rheumatic and musculoskeletal diseases: A systematic review of qualitative studies to inform the work of the OMERACT Foot and Ankle Working Group. Semin Arthritis Rheum. 2023;61:152212.

3.	Aouad K, Wendling D, Breban M, Dadoun S, Hudry C, Moltó A, et al. Exploring remission concept in axial spondyloarthritis through the perception of rheumatologists using vignettes and priority ratings. Rheumatology (Oxford). 2022;61(6):2603-8.

4.	Wendling D, Hecquet S, Fogel O, Letarouilly JG, Verhoeven F, Pham T, et al. 2022 French Society for Rheumatology (SFR) recommendations on the everyday management of patients with spondyloarthritis, including psoriatic arthritis. Joint Bone Spine. 2022;89(3):105344.

5.	Manara M, Prevete I, Marchesoni A, D'Angelo S, Cauli A, Zanetti A, et al. The Italian Society for Rheumatology recommendations for the management of axial spondyloarthritis. Reumatismo. 2021;73(2):71-88.

6.	Navarro-Compán V, Boel A, Boonen A, Mease P, Landewé R, Kiltz U, et al. The ASAS-OMERACT core domain set for axial spondyloarthritis. Semin Arthritis Rheum. 2021;51(6):1342-9.

7.	Baillet A, Romand X, Pflimlin A, Dalecky M, Claudepierrec P, Flipo RM, et al. Data to be collected for an optimal management of axial spondyloarthritis in daily practice: Proposal from evidence-based and consensual approaches. Joint Bone Spine. 2020;87(5):405-11.

8.	Kiltz U, Landewé RBM, van der Heijde D, Rudwaleit M, Weisman MH, Akkoc N, et al. Development of ASAS quality standards to improve the quality of health and care services for patients with axial spondyloarthritis. Ann Rheum Dis. 2020;79(2):193-201.

9.	Oude Voshaar MAH, Das Gupta Z, Bijlsma JWJ, Boonen A, Chau J, Courvoisier DS, et al. International Consortium for Health Outcome Measurement Set of Outcomes That Matter to People Living With Inflammatory Arthritis: Consensus From an International Working Group. Arthritis Care Res (Hoboken). 2019;71(12):1556-65.

10.	Schmalz G, Douglas D, Douglas D, Patschan S, Patschan D, Müller GA, et al. Oral health-related quality of life is associated with disease specific parameters in patients with ankylosing spondylitis. Clin Oral Investig. 2018;22(8):2889-96.

11.	Smolen JS, Schöls M, Braun J, Dougados M, FitzGerald O, Gladman DD, et al. Treating axial spondyloarthritis and peripheral spondyloarthritis, especially psoriatic arthritis, to target: 2017 update of recommendations by an international task force. Ann Rheum Dis. 2018;77(1):3-17.

12.	Kwan YH, Fong W, Tan VIC, Lui NL, Malhotra R, Østbye T, Thumboo J. A systematic review of quality-of-life domains and items relevant to patients with spondyloarthritis. Semin Arthritis Rheum. 2017;47(2):175-82.

13.	Martínez López JA, García Vivar ML, Cáliz R, Freire M, Galindo M, Hernández MV, et al. Recommendations for the evaluation and management of patients with rheumatic autoimmune and inflammatory diseases during the reproductive age, pregnancy, postpartum and breastfeeding. Reumatol Clin. 2017;13(5):264-81.

14.	Baillet A, Gossec L, Carmona L, Wit M, van Eijk-Hustings Y, Bertheussen H, et al. Points to consider for reporting, screening for and preventing selected comorbidities in chronic inflammatory rheumatic diseases in daily practice: a EULAR initiative. Ann Rheum Dis. 2016;75(6):965-73.

15.	Gossec L, Baillet A, Dadoun S, Daien C, Berenbaum F, Dernis E, et al. Collection and management of selected comorbidities and their risk factors in chronic inflammatory rheumatic diseases in daily practice in France. Joint Bone Spine. 2016;83(5):501-9.

16.	Millner JR, Barron JS, Beinke KM, Butterworth RH, Chasle BE, Dutton LJ, et al. Exercise for ankylosing spondylitis: An evidence-based consensus statement. Semin Arthritis Rheum. 2016;45(4):411-27.

17.	Boonen A, van Berkel M, Cieza A, Stucki G, van der Heijde D. Which aspects of functioning are relevant for patients with ankylosing spondylitis: results of focus group interviews. J Rheumatol. 2009;36(11):2501-11.

18.	Boonen A, van Berkel M, Kirchberger I, Cieza A, Stucki G, van der Heijde D. Aspects relevant for functioning in patients with ankylosing spondylitis according to the health professionals: a Delphi study with the ICF as reference. Rheumatology (Oxford). 2009;48(8):997-1002.

19.	Sidiropoulos PI, Hatemi G, Song IH, Avouac J, Collantes E, Hamuryudan V, et al. Evidence-based recommendations for the management of ankylosing spondylitis: systematic literature search of the 3E Initiative in Rheumatology involving a broad panel of experts and practising rheumatologists. Rheumatology (Oxford). 2008;47(3):355-61.

20.	Katz PP, Barber CEH, Duarte-García A, Garg S, Machua W, Rodgers W, et al. Development of the American College of Rheumatology Patient-Reported Outcome Quality Measures for Systemic Lupus Erythematosus. Arthritis Care Res (Hoboken). 2024;76(6):777-87.

21.	Fautrel B, Kedra J, Rempenault C, Juge PA, Drouet J, Avouac J, et al. 2024 update of the recommendations of the French Society of Rheumatology for the diagnosis and management of patients with rheumatoid arthritis. Joint Bone Spine. 2024;91(6):105790.

22.	Maica GL, Iannaccone C, Feathers V, Frits ML, Bykerk VP, Bingham CO, 3rd, et al. Understanding Differences in Patient Descriptions of Rheumatoid Arthritis Flares Using OMERACT Core Domains. J Rheumatol. 2023;50(7):889-94.

23.	Smolen JS, Landewé RBM, Bergstra SA, Kerschbaumer A, Sepriano A, Aletaha D, et al. EULAR recommendations for the management of rheumatoid arthritis with synthetic and biological disease-modifying antirheumatic drugs: 2022 update. Ann Rheum Dis. 2023;82(1):3-18.

24.	Coates LC, Soriano ER, Corp N, Bertheussen H, Callis Duffin K, Campanholo CB, et al. Group for Research and Assessment of Psoriasis and Psoriatic Arthritis (GRAPPA): updated treatment recommendations for psoriatic arthritis 2021. Nat Rev Rheumatol. 2022;18(8):465-79.

25.	Pallanti S, Porta F, Salerno L. Adult attention deficit hyperactivity disorder in patients with fibromyalgia syndrome: Assessment and disabilities. J Psychiatr Res. 2021;136:537-42.

26.	Mecoli CA, Park JK, Alexanderson H, Regardt M, Needham M, de Groot I, et al. Perceptions of Patients, Caregivers, and Healthcare Providers of Idiopathic Inflammatory Myopathies: An International OMERACT Study. J Rheumatol. 2019;46(1):106-11.

27.	Torre Alonso JC, Díaz Del Campo Fontecha P, Almodóvar R, Cañete JD, Montilla Morales C, Moreno M, et al. Recommendations of the Spanish Society of Rheumatology on treatment and use of systemic biological and non-biological therapies in psoriatic arthritis. Reumatol Clin (Engl Ed). 2018;14(5):254-68.

28.	Omair MA, Omair MA, Halabi H. Survey on management strategies of rheumatoid arthritis in Saudi Arabia: a Saudi Society for Rheumatology Initiative. Int J Rheum Dis. 2017;20(9):1185-92.

29.	Thumboo J, Wu L, Leung YY. Domains of quality of life affecting elderly patients with hand osteoarthritis: a qualitative study in the Asian perspective. Int J Rheum Dis. 2017;20(9):1105-19.

30.	Bacalao EJ, Greene GJ, Beaumont JL, Eisenstein A, Muftic A, Mandelin AM, et al. Standardizing and personalizing the treat to target (T2T) approach for rheumatoid arthritis using the Patient-Reported Outcomes Measurement Information System (PROMIS): baseline findings on patient-centered treatment priorities. Clin Rheumatol. 2017;36(8):1729-36.

31.	Gladman DD, Poulin Y, Adams K, Bourcier M, Barac S, Barber K, et al. Treating Psoriasis and Psoriatic Arthritis: Position Paper on Applying the Treat-to-target Concept to Canadian Daily Practice. J Rheumatol. 2017;44(4):519-34.

32.	Lackner A, Ficjan A, Stradner MH, Hermann J, Unger J, Stamm T, et al. It's more than dryness and fatigue: The patient perspective on health-related quality of life in Primary Sjögren's Syndrome - A qualitative study. PLoS One. 2017;12(2):e0172056.

33.	Yates M, Watts RA, Bajema IM, Cid MC, Crestani B, Hauser T, et al. EULAR/ERA-EDTA recommendations for the management of ANCA-associated vasculitis. Ann Rheum Dis. 2016;75(9):1583-94.

34.	Smolen JS, Breedveld FC, Burmester GR, Bykerk V, Dougados M, Emery P, et al. Treating rheumatoid arthritis to target: 2014 update of the recommendations of an international task force. Ann Rheum Dis. 2016;75(1):3-15.

35.	Regardt M, Basharat P, Christopher-Stine L, Sarver C, Björn A, Lundberg IE, et al. Patients' Experience of Myositis and Further Validation of a Myositis-specific Patient Reported Outcome Measure - Establishing Core Domains and Expanding Patient Input on Clinical Assessment in Myositis. Report from OMERACT 12. J Rheumatol. 2015;42(12):2492-5.

36.	Dejaco C, Singh YP, Perel P, Hutchings A, Camellino D, Mackie S, et al. 2015 recommendations for the management of polymyalgia rheumatica: a European League Against Rheumatism/American College of Rheumatology collaborative initiative. Arthritis Rheumatol. 2015;67(10):2569-80.

37.	Graf SW, Whittle SL, Wechalekar MD, Moi JH, Barrett C, Hill CL, et al. Australian and New Zealand recommendations for the diagnosis and management of gout: integrating systematic literature review and expert opinion in the 3e Initiative. Int J Rheum Dis. 2015;18(3):341-51.

38.	Dür M, Coenen M, Stoffer MA, Fialka-Moser V, Kautzky-Willer A, Kjeken I, et al. Do patient-reported outcome measures cover personal factors important to people with rheumatoid arthritis? A mixed methods design using the International Classification of Functioning, Disability and Health as frame of reference. Health Qual Life Outcomes. 2015;13:27.

39.	Bykerk VP, Lie E, Bartlett SJ, Alten R, Boonen A, Christensen R, et al. Establishing a core domain set to measure rheumatoid arthritis flares: report of the OMERACT 11 RA flare Workshop. J Rheumatol. 2014;41(4):799-809.

40.	Stamm T, Hieblinger R, Boström C, Mihai C, Birrell F, Thorstensson C, et al. Similar problem in the activities of daily living but different experience: a qualitative analysis in six rheumatic conditions and eight European countries. Musculoskeletal Care. 2014;12(1):22-33.

41.	Ranganath VK, Maranian P, Elashoff DA, Woodworth T, Khanna D, Hahn T, et al. Comorbidities are associated with poorer outcomes in community patients with rheumatoid arthritis. Rheumatology (Oxford). 2013;52(10):1809-17.

42.	Bartlett SJ, Hewlett S, Bingham CO, 3rd, Woodworth TG, Alten R, Pohl C, et al. Identifying core domains to assess flare in rheumatoid arthritis: an OMERACT international patient and provider combined Delphi consensus. Ann Rheum Dis. 2012;71(11):1855-60.

43.	Berthelot JM, De Bandt M, Morel J, Benatig F, Constantin A, Gaudin P, et al. A tool to identify recent or present rheumatoid arthritis flare from both patient and physician perspectives: the 'FLARE' instrument. Ann Rheum Dis. 2012;71(7):1110-6.

44.	Cunha-Miranda L, Costa L, Ribeiro JS. NEAR study: Needs and Expectations in Rheumatoid ARthritis - do we know our patients needs? Acta Reumatol Port. 2010;35(3):314-23.

45.	Mosca M, Tani C, Aringer M, Bombardieri S, Boumpas D, Brey R, et al. European League Against Rheumatism recommendations for monitoring patients with systemic lupus erythematosus in clinical practice and in observational studies. Ann Rheum Dis. 2010;69(7):1269-74.

46.	Sanderson T, Morris M, Calnan M, Richards P, Hewlett S. Patient perspective of measuring treatment efficacy: the rheumatoid arthritis patient priorities for pharmacologic interventions outcomes. Arthritis Care Res (Hoboken). 2010;62(5):647-56.

47.	Schoels M, Aletaha D, Smolen JS, Bijlsma JW, Burmester G, Breedveld FC, et al. Follow-up standards and treatment targets in rheumatoid arthritis: results of a questionnaire at the EULAR 2008. Ann Rheum Dis. 2010;69(3):575-8.

48.	van Hulst LT, Fransen J, den Broeder AA, Grol R, van Riel PL, Hulscher ME. Development of quality indicators for monitoring of the disease course in rheumatoid arthritis. Ann Rheum Dis. 2009;68(12):1805-10.

49.	Mease PJ, Arnold LM, Crofford LJ, Williams DA, Russell IJ, Humphrey L, et al. Identifying the clinical domains of fibromyalgia: contributions from clinician and patient Delphi exercises. Arthritis Rheum. 2008;59(7):952-60.

50.	Stamm TA, Nell V, Mathis M, Coenen M, Aletaha D, Cieza A, et al. Concepts important to patients with psoriatic arthritis are not adequately covered by standard measures of functioning. Arthritis Rheum. 2007;57(3):487-94.

51.	Zhang W, Doherty M, Arden N, Bannwarth B, Bijlsma J, Gunther KP, et al. EULAR evidence based recommendations for the management of hip osteoarthritis: report of a task force of the EULAR Standing Committee for International Clinical Studies Including Therapeutics (ESCISIT). Ann Rheum Dis. 2005;64(5):669-81.

**Supplementary File S4.** Candidate domains to be considered for the ASAS core set for the assessment of axial spondyloarthritis in clinical practice, with the corresponding definitions

**Disease manifestations of axSpA**

Disease activity

Disease activity covers the level of inflammatory disease attributable to the disease, for example, based on the signs and symptoms that a patient presents with.

Pain

Pain includes overall pain, peripheral pain (pain in the hands and feet, wrists, elbows, shoulders, ankles, and knees) and/or spinal pain (pain in the neck and spine) experienced throughout the day and at night. The sensation of pain (unpleasant feeling indicating potential or actual damage to some body part or throughout the body), as well as pain intensity (how much pain is present) and duration (how long pain is present) are relevant aspects. A distinction can be made between pain attributed to inflammatory and non-inflammatory processes.

Morning stiffness

Morning stiffness refers to the presence and duration of a feeling of stiffness in the back upon getting up in the morning, which influences the ability to move about.

Fatigue

Fatigue describes the overall feeling of tiredness and/or lack of energy, as well as the inability to optimally use mental or physical capacity due to this feeling.

Sleep

Sleep includes the quality of sleep (e.g. ability to sleep through the night, waking up feeling well-rested), as well as any sleep problems (e.g. trouble falling asleep, waking up numerous times a night) caused by complaints.

Peripheral manifestations

Peripheral manifestations include peripheral arthritis, enthesitis, and dactylitis.

Extra-musculoskeletal manifestations

Extra-musculoskeletal manifestations include uveitis, inflammatory bowel disease, and psoriasis. These are frequently occurring in patients with axial spondyloarthritis and belong to the disease spectrum.

**Physical consequences of disease**

Structural damage

Structural damage includes objective, irreversible damage to the spine and sacroiliac joints, peripheral joints (hands and feet, elbows, wrists, ankles, and knees), and root joints (shoulders and hips).

Mobility

Mobility describes the range of motion and ease of movement of a joint. This includes mobility of a single joint or several joints, such as of the spine (e.g. how much the spine can bend forward) and neck (e.g. how many degrees the neck can turn), as well as the ability to perform functions such as rolling over, sitting, standing, and walking.

Physical appearance

Physical appearance refers to the outward characteristics of the body. Deformities in physical appearance in axial spondyloarthritis may include postural or positional abnormalities and visible changes to the skin or nails.

**Consequences of disease on functioning and participation**

Overall functioning and health

Overall functioning and health refers to the general wellbeing of an individual in the context of their physical, mental, and social aspects of life. A prominent component is quality of life, defined as the perceived standard of health, comfort, and happiness experienced by an individual in their daily life. Health-related quality of life (HRQoL) is an assessment of how the wellbeing of an individual may be affected over time by a disease, disability, or disorder. In assessing HRQoL, participation at work, home and leisure, overall wellbeing, daily function, social support from family and friends, interpersonal relationships, and social roles may be considered. Also included are any impairments experienced during the day as a result of sleep problems.

Physical functioning

Physical functioning is defined as the ability to carry out activities that require physical capability, ranging from self-care (activities of daily living) to more vigorous activities that require increasing degrees of mobility, strength, or endurance. Physical performance (degree of physical activity, strength, balance, and endurance), as well as physical difficulty (any problems with physical activity resulting from impairment, any activity limitations and participation restrictions) and the ability to transfer oneself from one place to another (e.g. walking, cycling) are relevant aspects.

Cognitive functioning

Cognitive functioning refers to a range of mental processes relating to the acquisition, storage, manipulation, and retrieval of information. All forms of knowing and awareness are included, such as perceiving, conceiving, remembering, reasoning, judging, imagining, and problem solving.

Emotional functioning

Emotional functioning includes affect (e.g. sadness, happiness, love, fear, anger, hate, tension, anxiety, depression, joy, sorrow), and disorders hereof (e.g. lability of emotion and flattening of affect). Also relevant is the emotional impact of disease, relating to (emotional) coping and acceptance.

Sexual and reproductive health*

Sexual and reproductive health refers to the physical and psychological wellbeing and capacity of an individual in relation to engaging in intimate relationships, as well as participating in reproductive planning, including the potential to conceive, carry, and give birth to children, if desired.

**Consequences of disease on activities and participation**

Work and employment

Work and employment includes the ability to work and be productive at work, as well as to engage in all aspects of work (e.g. seeking employment and getting a job, doing the required tasks of the job, attending work on time as required, supervising other workers or being supervised, and performing required tasks alone or in groups).

Social functioning

Social functioning encompasses the ability of an individual to interact effectively and appropriately with others in various social contexts, as well as the capacity to build and maintain relationships and fulfil social roles. It includes functioning in both informal settings (e.g. family and family planning, friendships, relationships with informal caregivers) and formal settings (e.g. relationships with healthcare professionals), and the reliance an individual has on these social constructs.

**Personal and treatment aspects**

Lifestyle

Lifestyle refers to the habits, routines, choices, values, and behaviours of an individual makes on a daily basis, which may, for example, manifest in hobbies. Aspects of lifestyle that may affect health, wellbeing, and disease management are also included, for example, diet and nutrition, levels of physical activity, and intoxications (e.g. smoking and recreational drug use).

Comorbidities

Comorbidities are medical conditions that co-exist alongside the primary diagnosis. Examples include cardiovascular disease, malignancies, and osteoporosis.

Treatment aspects

Treatment aspects encompasses the assessment of current pharmacological treatments (e.g. adherence and safety) and non-pharmacological treatments, as well as the nature of healthcare utilisation by an individual as a whole.

Self-management*

Self-management refers to the ability of a patient to participate in the management of their disease, including the physical and psychosocial impact thereof.

Patient-specific factors

Patient-specific factors include the personal considerations to be evaluated in the treatment trajectory of an individual patient. Examples include specific concerns a patient may present with, particular expectations or wishes held by a patient, disease knowledge and awareness, health literacy, and time investment in the disease management process.

* Domain was added later to a revised version of the first Delphi round based on participant suggestions.

Definitions adapted from: Boel A, Navarro-Compán V, Boonen A, Mease P, Kiltz U, Dougados M, et al. Domains to Be Considered for the Core Outcome Set of Axial Spondyloarthritis: Results From a 3-round Delphi Survey. J Rheumatol. 2021;48(12):1810-4.

**Supplementary File S5.** Characteristics of participants who completed the first Delphi round

| Variable | ASAS members (n=156) | Non-ASAS rheumatologists (n=173) | Patients (n=167) |
| --- | --- | --- | --- |
| Female sex | 59 (38) | 94 (54) | 100 (60) |
| Age <50 years | 68 (44) | 128 (74) | 99 (59) |
| Continent of origin  Africa  Asia  Europe  North America  Oceania  South America | 4 (3)  27 (17)  91 (58)  19 (12)  1 (1)  14 (9) | 8 (5)  42 (24)  98 (57)  4 (2)  0 (0)  21 (12) | 9 (5)  29 (17)  22 (13)  45 (27)  6 (4)  56 (34) |
| Primary place of work/treatment  University hospital  General hospital  Private practice  Other | 128 (82)  7 (4)  11 (7)  10 (6) | 90 (52)  44 (25)  32 (18)  7 (4) | 25 (15)  49 (29)  77 (46)  16 (10) |
| Primary work function  (Non-)practicing rheumatologist  Other HCP  Researcher | 136 (87)  8 (5)  12 (8) | 173 (100)  NA  NA | NA |
| Work experience as MD <20 years* | 51 (38) | 121 (70) | NA |
| Number of patients with axSpA treated per year*  <100  100-300  >300 | 24 (18)  63 (47)  47 (35) | 95 (55)  60 (35)  18 (10) | NA |
| Ancillary HCPs available in workplace* | 63 (47) | 70 (40) | NA |
| Disease duration of axSpA <20 years | NA | NA | 124 (74) |
| Follow-up frequency per year for axSpA  <1 visits  1-2 visits  3-4 visits  >4 visits | NA | NA | 17 (10)  72 (43)  48 (29)  30 (18) |
| Patient global assessment of disease activity ≤5/10 | NA | NA | 77 (46) |
| Patient global assessment of disease activity, mean (SD) | NA | NA | 5.7 (2.5) |
| All values presented as n (%), unless otherwise indicated. Age, work experience, number of patients treated, disease duration, and follow-up frequency were collected on a categorical scale.  * Percentage for ASAS members was calculated only including practicing rheumatologists (n=134/156 participating ASAS members).  ASAS: Assessment of SpondyloArthritis international Society; axSpA: axial spondyloarthritis; HCP: healthcare professional; MD: medical doctor; NA: not applicable. | | | |

**Supplementary Figure S2.** Geographical distribution of participants. The 46 countries represented by at least one ASAS member or non-ASAS rheumatologist (left) and 17 countries represented by at least one patient (right) in the Delphi survey are coloured.

ASAS: Assessment of SpondyloArthritis international Society.

**Supplementary File S6.** Characteristics of ASAS members who completed the first Delphi round, stratified by (non-)participation in subsequent rounds to illustrate attrition across the Delphi survey

| Variable | Total participants round 1 (n=156) | Non-responders round 2 (n=14/156)^†^ | Non-responders round 3 (n=31/156)^†^ | Responders to all three planned rounds (n=124/156) |
| --- | --- | --- | --- | --- |
| Female sex | 59 (38) | 3 (21) | 12 (39) | 47 (38) |
| Age <50 years | 68 (44) | 4 (29) | 14 (45) | 54 (44) |
| Continent of origin  Africa  Asia  Europe  North America  Oceania  South America | 4 (3)  27 (17)  91 (58)  19 (12)  1 (1)  14 (9) | 0 (0)  2 (14)  5 (36)  5 (36)  0 (0)  2 (14) | 0 (0)  3 (10)  18 (58)  7 (23)  0 (0)  3 (10) | 4 (3)  24 (19)  72 (58)  12 (10)  1 (1)  11 (9) |
| Primary place of work  University hospital  General hospital  Private practice  Other | 128 (82)  7 (4)  11 (7)  10 (6) | 9 (64)  2 (14)  1 (7)  2 (14) | 24 (77)  2 (6)  1 (3)  4 (13) | 103 (83)  5 (4)  10 (8)  6 (5) |
| Primary work function  (Non-)practicing rheumatologist  Other HCP  Researcher | 136 (87)  8 (5)  12 (8) | 11 (79)  3 (21)  0 (0) | 24 (77)  6 (19)  1 (3) | 111 (90)  2 (2)  11 (9) |
| Work experience as MD <20 years* | 51/134 (38) | 3/9 (33) | 10/22 (45) | 41/111 (37) |
| Number of patients with axSpA treated per year*  <100  100-300  >300 | 24/134 (18)  63/134 (47)  47/134 (35) | 3/9 (33)  2/9 (22)  4/9 (44) | 7/22 (32)  7/22 (32)  8/22 (36) | 17/111 (15)  56/111 (50)  38/111 (34) |
| Ancillary HCPs available in workplace* | 63/134 (47) | 4/9 (44) | 11/22 (50) | 52/111 (47) |
| All values presented as n (%). Age, work experience, and number of patients treated were collected on a categorical scale.  * Percentage was calculated only including practicing rheumatologists.  ^†^ All ASAS members (n=201) were invited for each Delphi round, as non-participation in earlier rounds did not preclude being invited for subsequent rounds. Consequently, some participants completed the second round (n=12/154 total participants) and third round (n=17/142 total participants) despite not having participated in the first round. To illustrate attrition across the Delphi survey, however, the participant numbers presented in the table are restricted to those who completed the first round.  ASAS: Assessment of SpondyloArthritis international Society; axSpA: axial spondyloarthritis; HCP: healthcare professional; MD: medical doctor. | | | | |

**Supplementary File S7.** Characteristics of non-ASAS member rheumatologists who completed the first Delphi round, stratified by (non-)participation in subsequent rounds to illustrate attrition across the Delphi survey

| Variable | Total participants round 1 and invited for subsequent rounds (n=173) | Non-responders round 2 (n=74/173) | Non-responders round 3 (n=91/173)* | Responders to all three planned rounds (n=67/173) |
| --- | --- | --- | --- | --- |
| Female sex | 94 (54) | 41 (55) | 48 (53) | 35 (52) |
| Age <50 years | 128 (74) | 55 (74) | 69 (76) | 48 (72) |
| Continent of origin  Africa  Asia  Europe  North America  Oceania  South America | 8 (5)  42 (24)  98 (57)  4 (2)  0 (0)  21 (12) | 5 (7)  19 (26)  34 (46)  2 (3)  0 (0)  14 (19) | 4 (4)  20 (22)  51 (56)  3 (3)  0 (0)  13 (14) | 3 (4)  18 (27)  40 (60)  1 (1)  0 (0)  5 (7) |
| Primary place of work  University hospital  General hospital  Private practice  Other | 90 (52)  44 (25)  32 (18)  7 (4) | 42 (57)  10 (14)  19 (26)  3 (4) | 50 (55)  18 (20)  20 (22)  3 (3) | 34 (51)  24 (36)  6 (9)  3 (4) |
| Work experience as MD <20 years | 121 (70) | 54 (73) | 63 (69) | 47 (70) |
| Number of patients with axSpA treated per year  <100  100-300  >300 | 95 (55)  60 (35)  18 (10) | 43 (58)  28 (38)  3 (4) | 48 (53)  35 (38)  8 (9) | 40 (60)  18 (27)  9 (13) |
| Ancillary HCPs available in workplace | 70 (40) | 28 (38) | 34 (37) | 30 (45) |
| All values presented as n (%). Age, work experience, and number of patients treated were collected on a categorical scale.  * All non-ASAS rheumatologists who participated in the first Delphi round (n=173) were invited for each following round, as non-participation in earlier rounds did not preclude being invited for subsequent rounds. Consequently, some participants completed the third round without having participated in the second round (n=15/82 total participants).  ASAS: Assessment of SpondyloArthritis international Society; axSpA: axial spondyloarthritis; HCP: healthcare professional; MD: medical doctor. | | | | |

**Supplementary File S8.** Characteristics of patients who completed the first Delphi round, stratified by (non-)participation in subsequent rounds to illustrate attrition across the Delphi survey

| Variable | Total participants round 1 and invited for subsequent rounds (n=167) | Non-responders round 2 (n=75/167) | Responders to both planned rounds for patients (n=92/167) |
| --- | --- | --- | --- |
| Female sex | 100 (60) | 46 (61) | 54 (59) |
| Age <50 years | 99 (59) | 53 (71) | 46 (50) |
| Continent of origin  Africa  Asia  Europe  North America  Oceania  South America | 9 (5)  29 (17)  22 (13)  45 (27)  6 (4)  56 (34) | 2 (3)  13 (17)  9 (12)  16 (21)  3 (4)  32 (43) | 7 (8)  16 (17)  13 (14)  29 (32)  3 (3)  24 (26) |
| Primary place of treatment  University hospital  General hospital  Private practice  Other | 25 (15)  49 (29)  77 (46)  16 (10) | 10 (13)  25 (33)  32 (43)  8 (11) | 15 (16)  24 (26)  45 (49)  8 (9) |
| Disease duration of axSpA <20 years | 124 (74) | 56 (75) | 68 (74) |
| Follow-up frequency per year for axSpA  <1 visit  1-2 visits  3-4 visits  >4 visits | 17 (10)  72 (43)  48 (29)  30 (18) | 5 (7)  32 (43)  27 (36)  11 (15) | 12 (13)  40 (43)  21 (23)  19 (21) |
| Patient global assessment of disease activity ≤5/10 | 77 (46) | 32 (43) | 45 (49) |
| Patient global assessment of disease activity, mean (SD) | 5.7 (2.5) | 5.9 (2.5) | 5.6 (2.5) |
| Survey language  English  Spanish | 116 (69)  51 (31) | 44 (59)  31 (41) | 72 (78)  20 (22) |
| All values presented as n (%), unless otherwise stated. Age, disease duration, and follow-up frequency were collected on a categorical scale.  axSpA: axial spondyloarthritis. | | | |

**Supplementary File S9.** Additional results from the first Delphi round

**Consolidated results from the rating and ranking questions**

| Domains | **Routine outpatient visits** | | | **Extended outpatient visits** | | |
| --- | --- | --- | --- | --- | --- | --- |
|  | ASAS members | Non-ASAS | Patients | ASAS members | Non-ASAS | Patients |
| **Disease activity** |  |  |  |  |  |  |
| **Pain** |  |  |  |  |  |  |
| **Morning stiffness** |  |  |  |  |  |  |
| **Fatigue** |  |  |  |  |  |  |
| Sleep |  |  |  |  |  |  |
| **EMMs** |  |  |  |  |  |  |
| **Peripheral manifestations** |  |  |  |  |  |  |
| **Structural damage** |  |  |  |  |  |  |
| **Mobility** |  |  |  |  |  |  |
| Physical appearance |  |  |  |  |  |  |
| **Overall functioning and health** |  |  |  |  |  |  |
| **Physical functioning** |  |  |  |  | **†** |  |
| Cognitive functioning |  |  |  |  |  |  |
| Emotional functioning |  |  |  |  |  |  |
| Sexual and reproductive health* | NA |  |  | NA |  |  |
| Work and employment |  |  |  |  |  |  |
| Social functioning |  |  |  |  |  |  |
| Lifestyle |  |  |  |  |  |  |
| **Comorbidities** |  |  |  |  |  |  |
| **Treatment aspects** |  |  |  |  |  |  |
| **Self-management*** | NA |  |  | NA | **†** |  |
| **Patient-specific factors** |  |  |  |  |  |  |

All domains included in the first Delphi round are shown. For each stakeholder group, the five highest-ranked domains by weighted scoring within that group, that were also rated as relevant by ≥70% of participants, are coloured green. Domains that did not fulfil these criteria are coloured red. Domains that fulfilled these criteria in at least one stakeholder group, and were therefore either pre-selected or carried forward to the second round, are bolded.

* Domain was added later to a revised version of the first Delphi round based on participant suggestions. Results presented are therefore only based on participants who completed the revised version (57 of 173 participating non-ASAS rheumatologists and all 167 patients).

^†^ The ‘physical functioning’ domain fulfilled the selection criteria only in the group of non-ASAS rheumatologists who completed the original version of the survey containing 20 candidate domains (n=116/173), and the ‘self-management’ domain only in the group who completed the revised version containing 22 candidate domains (n=57/173).

ASAS: Assessment of SpondyloArthritis international Society; EMM: extra-musculoskeletal manifestation; NA: not available in survey; non-ASAS: non-ASAS member rheumatologists.

**Supplementary File S10.** Additional results from the second Delphi round

**Consolidated results from the ranking questions**

Results of the second Delphi round for ASAS members and non-ASAS rheumatologists combined (n=253)

| Domain | **Routine outpatient visits** | | | **Extended outpatient visits** | | | **Cumulative** | |
| --- | --- | --- | --- | --- | --- | --- | --- | --- |
|  | Ranking | *N* | % | Ranking | *N* | % | *N* | % |
| **Disease activity** | #1 | *Pre-selected** | | NA | | | *Pre-selected** | |
| **Pain** | #2 | *Pre-selected** | | NA | | | *Pre-selected** | |
| **Peripheral man.** | #3 | 130 | 51% | NA | | | 130 | 51% |
| **EMMs** | #4 | 137 | 54% | #6 | 42 | 17% | 179 | 71% |
| **Treatment aspects** | #5 | 146 | 58% | #8 | 30 | 12% | 176 | 70% |
| **Overall functioning** | #6 | 44 | 17% | #4 | 104 | 41% | 148 | 58% |
| Mobility | #7 | 12 | 5% | #5 | 80 | 32% | 92 | 36% |
| Morning stiffness | #8 | 11 | 4% | NA | | | 11 | 4% |
| Fatigue | #9 | 11 | 4% | NA | | | 11 | 4% |
| **Structural damage** | NA | | | #1 | *Pre-selected** | | *Pre-selected** | |
| **Comorbidities** | NA | | | #2 | 157 | 62% | 157 | 62% |
| Physical functioning | NA | | | #3 | 100 | 40% | 100 | 40% |
| Self-management | NA | | | #7 | 43 | 17% | 43 | 17% |

*N* indicates the number of participants who included the domain in their ranking of the five most relevant domains. Domains that were among the five highest-ranked by weighted scoring for routine and/or extended visits and included in the rankings of a cumulative simple majority of participants (>50% across routine and extended visits) are bolded.

* Domain was pre-selected in the first round and therefore not available for voting in the second round.

EMMs: extra-musculoskeletal manifestations; Man: manifestations.

Results of the second Delphi round for patients (n=92)

| Domain | **Routine outpatient visits** | | | **Extended outpatient visits** | | | **Cumulative** | |
| --- | --- | --- | --- | --- | --- | --- | --- | --- |
|  | Ranking | *N* | % | Ranking | *N* | % | *N* | % |
| **Disease activity** | #1 | *Pre-selected** | | NA | | | *Pre-selected** | |
| **Pain** | #2 | *Pre-selected** | | NA | | | *Pre-selected** | |
| Peripheral man. | #8 | 18 | 20% | NA | | | 18 | 20% |
| EMMs | #5 | 23 | 25% | #8 | 16 | 17% | 39 | 42% |
| **Treatment aspects** | #4 | 23 | 25% | #5 | 24 | 26% | 47 | 51% |
| **Overall functioning** | #3 | 31 | 34% | #6 | 18 | 20% | 49 | 53% |
| Mobility | #9 | 10 | 11% | #7 | 19 | 21% | 29 | 32% |
| Morning stiffness | #6 | 15 | 16% | NA | | | 15 | 16% |
| Fatigue | #7 | 20 | 22% | NA | | | 20 | 22% |
| **Structural damage** | NA | | | #1 | *Pre-selected** | | *Pre-selected** | |
| Comorbidities | NA | | | #2 | 41 | 45% | 41 | 45% |
| Physical functioning | NA | | | #3 | 36 | 39% | 36 | 39% |
| Self-management | NA | | | #4 | 36 | 39% | 36 | 39% |

*N* indicates the number of participants who included the domain in their ranking of the five most relevant domains. Domains that were among the five highest-ranked by weighted scoring for routine and/or extended visits and included in the rankings of a cumulative simple majority of participants (>50% across routine and extended visits) are bolded.

* Domain was pre-selected in the first round and therefore not available for voting in the second round.

EMMs: extra-musculoskeletal manifestations; Man: manifestations.
